# Supplementary material for: SINDy-RL for interpretable and efficient model-based reinforcement learning
Source: Nat Commun. 2025 Nov 28;16:10714. doi: 10.1038/s41467-025-65738-4 (PMC12663201; doi:10.1038/s41467-025-65738-4)
Supplement: Supplementary file 1 — Supplementary Information [file 41467_2025_65738_MOESM1_ESM.pdf]

Supplementary Information

SINDy-RL: Interpretable and Efficient Model-Based Reinforcement Learning

Nicholas Zolman[*1,5], Christian Lagemann[1], Urban Fasel[2], J. Nathan Kutz[3,4], and Steven L. Brunton[1]

[1]*Department of Mechanical Engineering, University of Washington, Seattle, WA 98195, USA*
[2]*Department of Aeronautics, Imperial College, London SW7 2AZ, United Kingdom*
[3]*Department of Applied Mathematics, University of Washington, Seattle, WA 98195*
[4]*Department of Electrical and Computer Engineering, University of Washington, Seattle, WA 98195*
[5]*Data Science and Artificial Intelligence Department, The Aerospace Corporation, El Segundo, CA 90245*

Contents

1 Extended Introduction **2**

2 Extended Background **4**
2A Reinforcement Learning 4
2B Sparse Dictionary Learning 5

3 Environments **8**
3A Swing-up. 8
3B Swimmer-v4 9
3C Cylinder 9
3D Pinball 10
3E 3D Airfoil 11
3E.1 Numerical Setup 11
3E.2 Grid Resolution and Refinement Strategy 12
3E.3 Flow Conditions and Gust Implementation 12

4 DRL Training and Benchmarking **15**
4A MBRL Comparison—Swing-Up 16
4B Population-Based Training—Swimmer-v4 18
4C Cylinder Benchmark 19
4D Pinball Benchmark 20
4E 3D Airfoil Benchmark 21

5 Dictionary Dynamics **22**
5A Swing-up 22
5B Swimmer-v4 24
5C Cylinder 24
5D Pinball 25
5E 3D Airfoil 27

[*]Corresponding author (nzolman@uw.edu)

6 Dictionary Rewards 28
6A Swing-up 28
6B Swimmer-v4 28
6C Cylinder 28
6D Pinball 29
6E 3D Airfoil 31

7 Dictionary Policy 32
7A Policy Sampling Study 32
7B Swing-up 33
7C Swimmer-v4 34
7D Cylinder 35
7E Pinball 37
7F 3D Airfoil 38

1 Extended Introduction

Much of the success of modern technology can be attributed to our ability to control dynamical systems: designing safe biomedical implants for homeostatic regulation, gimbaling rocket boosters for reusable launch vehicles, operating power plants and power grids, industrial manufacturing, among many other examples. Over the past decade, advances in machine learning and optimization have rapidly accelerated our capabilities to tackle complicated data-driven tasks—particularly in the fields of computer vision [1] and natural language processing [2]. Reinforcement learning (RL) is at the intersection of both machine learning and optimal control, and the core ideas of RL date back to the infancy of both fields. By interacting with an environment and receiving feedback about its performance on a task through a reward, an RL agent iteratively improves a control policy. Deep reinforcement learning (DRL), in particular, has shown promise for uncovering control policies in complex, high-dimensional spaces [3–11]. DRL has been used to achieve super-human performance in games [12–16] and drone racing [17], to control the plasma dynamics in a tokamak fusion reactor [18], to discover novel drugs [19], and for many applications in fluid mechanics [20–30]. However, these methods rely on neural networks and typically suffer from three major drawbacks: (1) they are infeasible to train for many applications because they require millions—or even billions [16]—of interactions with the environment; (2) they are challenging to deploy in resource-constrained environments (such as embedded devices and micro-robotic systems) due to the size of the networks and need for specialized software; and (3) they are "black-box" models that lack interpretability, making them untrustworthy to operate in safety-critical systems or high-consequence environments. In this work, we seek to create interpretable and generalizable reinforcement learning methods that are also more sample efficient via sparse dictionary learning.

Sample-Efficient DRL. There has been significant research into reducing the amount of experience needed to train RL policies. Offline RL seeks to train policies on a pre-collected dataset as opposed to collecting new experience in an environment [31]. When the dataset consists of "expert" trajectories, imitation learning approaches, such as behavior cloning, have been successful in approximating the expert policy using offline RL, which has been widely used for autonomous robotic and vehicle systems [32–34]. Experience replay is a popular method to bridge offline and online RL, where policies are continuously trained on new and past experience using replay buffers [35, 36]. Hindsight experience replay takes previous low-scoring trials and alters them to be high-scoring by changing the goal, thus improving learning rates [37]. In contrast, transfer learning takes embodied knowledge from a similar, pre-trained task and uses it to initialize a new task—typically reusing many weights of a neural network and fine-tuning on the new task [38]. To increase the likelihood of transfer learning being successful, researchers have trained DRL policies to interact with many different (but similar) tasks or environments using meta-learning [39, 40].

Surrogate environments are perhaps the most common way to reduce the number of interactions in an environment. Whether it's a simulation of a physical environment or a low-fidelity approximation of a high-fidelity model, one can greatly reduce the number of agent interactions by training almost entirely in the surrogate. For instance, researchers first trained agents in a simulation before deploying the agent to a real tokamak [18]. Because surrogates are only approximations of the ground-truth environments, researchers have also explored "sim2real" approaches that transfer learned experience from a simulated environment to a real environment [41]. Direct access to the dynamics can also be exploited by model-based reinforcement learning (MBRL) which can be used to calculate optimal control using the Hamilton-Jacobi-Bellman equations or localized planning [42]. For example, model-predictive control (MPC) methods are frequently used in modern control to plan and execute optimal controls over finite-time horizons [43].

However, there are many cases where a surrogate model—such as a low-fidelity simulation—does not exist and the dynamics must be learned. Dyna-style MBRL algorithms iteratively switch between learning and improving a surrogate model of the environment and training model-free policies inside the surrogate environment by generating "imaginary" experience [44]. For physical environments, this iterative process of learning a surrogate model is particularly important because there may be regions of the environment that are impossible to access without a sophisticated controller—such as the unstable equilibrium of the inverted pendulum that starts in the stable, downward position. Deep MBRL algorithms have shown significant promise for reducing sample complexity on benchmark environments [45] by simultaneously training neural network models of the environment. For example, model-based meta-policy optimization (MB-MPO) [46] trains an ensemble of neural network models for the dynamics that can be used to learn policies capable of quickly adapting to different members of the ensemble. Although neural network models have recently become popular and are gaining wide adoption over traditional modeling methods, they are still overparameterized, data-inefficient, and uninterpretable. In this work, we examine MBRL using lightweight, sparse dictionary models [47] that are fast to train on limited data, convenient for estimating uncertainty, and provide an interpretable symbolic representation by construction.

Sparse Dictionary Learning. Sparse dictionary learning is a type of symbolic regression that learns a representation of a function as a sparse linear combination of pre-chosen candidate dictionary (or "library") functions. The dictionary functions, such as polynomials or trigonometric functions, are chosen based on domain expertise and are evaluated on input data. Sparse learning has become a popular method for discovering dynamics, as in the sparse identification of nonlinear dynamics (SINDy) algorithm [47], which models the vector field of a dynamical system as a sparse linear combination of dictionary terms. The sparse symbolic representation of the model lends itself naturally to interpretation and model analysis—especially for physical systems where the dictionary terms have physical significance [48–54]. SINDy has been extensively used to develop reduced-order models [55–63] and turbulence closures [64–67] in fluid mechanics. These methods have been extended to PDEs [68], simultaneously discovering coordinates from high-dimensional representations [69], and promoting stability [70]. Moreover there has been significant progress in improving SINDy techniques for the high-noise, low-data limit [71–76]. In particular, constructing an ensemble of SINDy models (E-SINDy) has been shown to provide an efficient and robust way to learn models from limited and noisy data [77] with accurate uncertainty quantification (UQ) [78, 79]. In addition to the inherent interpretability of a sparse symbolic model, quantifying uncertainty provides additional trust in the algorithm and is useful for sensitivity analyses.

Importantly, SINDy has been extended to systems with actuation and control [80, 81] and used for designing model predictive control laws [81–83]. SINDy methods are incredibly efficient—both for model creation and deployment—making them promising for both online learning and control in resource-constrained operations. In particular, SINDy has been used for online simultaneous dynamics discovery and optimal control design [84, 85]. Finally, recent work has suggested leveraging a single SINDy model to accelerate

DRL, and has demonstrated its use on simple DRL benchmarks [86]. In this work, we generalize this DRL framework to include ensembles of dictionary models to accelerate learning in the low-data limit and quantify uncertainty.

2 Extended Background

In this work, we consider control systems that evolve according to a governing differential equation

$$\frac{d}{dt}\mathbf{x} = f(\mathbf{x}, \mathbf{u})$$

or discrete-time update equation

$$\mathbf{x}_{k+1} = f(\mathbf{x}_k, \mathbf{u}_k)$$

where the state $\mathbf{x} \in \mathbb{R}^m$ and control input $\mathbf{u} \in \mathbb{R}^l$ are continuously defined variables. However, reinforcement learning is a more general framework [7, 87] and can be applied to a wider class of systems, such as board games with discrete action and/or observation spaces.

2A Reinforcement Learning

Reinforcement learning comprises a family of methods where an agent interacts in an environment, $\mathcal{E}$, through sequences of different states, $\mathbf{x}_n$, by taking actions $\mathbf{u}_n$ *. The agent interacts in the environment by sampling an action from a *policy* at each state $\mathbf{u}_n \sim \pi(\mathbf{x}_n)$ and executing it in the environment, producing a new state $\mathbf{x}_{n+1}$ and reward r_n—a signal indicating how well the agent performed on that step. We define the value function, V_π to be the expected future return for taking actions from the policy π:

$$V_\pi(\mathbf{x}) = \mathbb{E}\left(\sum_{k=0}^{\infty} \gamma^k r_k \middle| \mathbf{x}_0 = \mathbf{x}\right) \tag{1}$$

where $0 < \gamma \leq 1$ is the *discount rate* that weights earlier rewards to be worth more than later ones, and the expectation is taken when either the policy or environment are stochastic. RL methods seek to find a policy that maximizes the value function, i.e.

$$\pi^*(\mathbf{x}) = \underset{\pi}{\operatorname{argmax}}\, V_\pi(\mathbf{x}). \tag{2}$$

This can become a very challenging optimization problem, especially in the case of high-dimensional state-spaces, continuous action spaces, and nonlinear dynamics. Deep reinforcement learning (DRL) has made significant progress in addressing these problems by directly parameterizing functions as deep neural networks, such as the policy $\pi(\mathbf{x}) \approx \pi_\phi(\mathbf{x})$, and training on collected experiences. Supplementary Figure 1 shows a simplified schematic of DRL training and agent interaction.

Model-free DRL methods optimize control policies in an environment without any explicit model of how the environment will transition between states, and have gained popularity because of the ease of application and flexibility of the framework to a given problem. Model-free DRL approaches, however, generally require an enormous number of training experience. This is in contrast to *model-based* reinforcement learning (MBRL) methods which have shown to be substantially more sample-efficient by exploiting a model of the environment [45]. Classical model-based approaches, such as model-predictive control (MPC), exploit a model to plan optimal trajectories over a finite time-horizon. More generally, shooting algorithms [88]

*It has become popular to refer to actions as a_n and states as s_n in the reinforcement literature; however, we use u_n and x_n to better align with the control and dynamics communities.

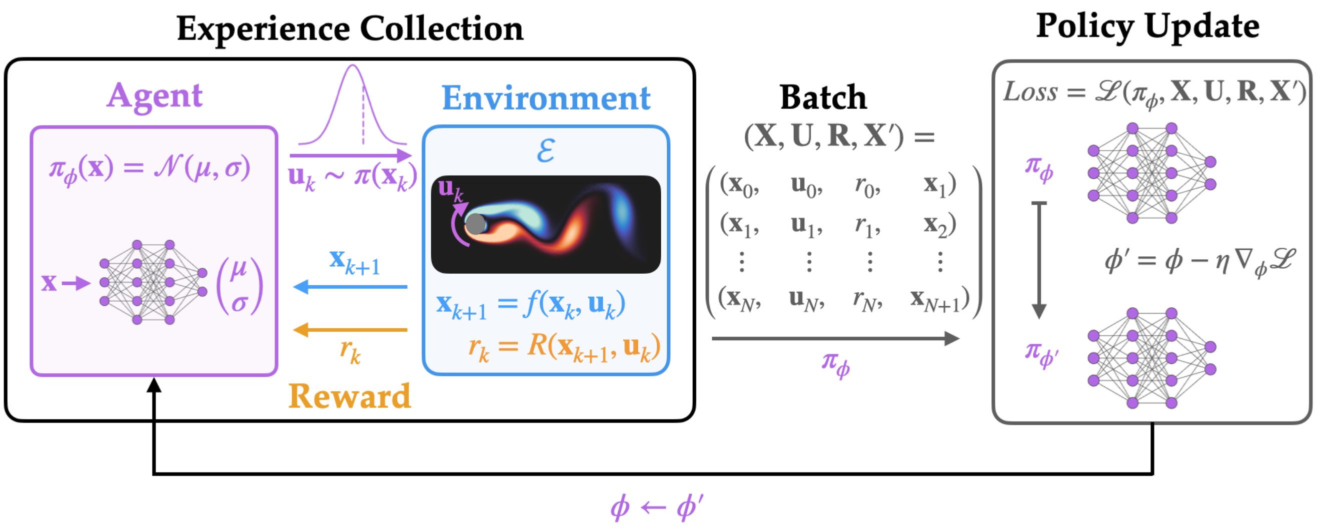

Supplementary Figure 1: An overview of deep reinforcement learning. *Left*: An agent collects experience by sampling an action from the policy distribution (parameterized by a deep policy network) which is executed in the environment. The environment provides a new state and reward based on the performance. *Right*: A batch of experience, collected in (state, action, reward, new state) sequences, is fed to the policy optimization, where the loss is computed. The neural network parameters ϕ are updated using a gradient-based optimization.

are popular approaches for solving these optimization problems, and neural networks have become widely adopted for representing complicated environment dynamics [89, 90]. Other MBRL approaches optimize V_π directly by calculating the gradients analytically with respect to the policy actions or exploiting modern auto-differentiation software to back-propagate gradients through a parameterized policy [91–94]. Finally, in order to exploit the success of model-free approaches while reducing the total number of interactions in a ground-truth environment, Dyna-style RL methods learn a surrogate model of the environment, such as linear models [95], neural networks [46], dynamic mode decomposition or Koopman models [96, 97], and train agents to interact in this approximation of the environment.

2B Sparse Dictionary Learning

Sparse dictionary learning methods are a form of symbolic regression used to uncover interpretable representations of nonlinear functions from available data. Many symbolic regression algorithms utilize evolutionary or genetic algorithms to generate functions as the combinations of some candidate library functions through elementary mathematical operations (e.g. addition, multiplication, and composition) [98–100]. These methods can be very expressive; however, the search space is typically vast, and since they rely on gradient-free algorithms that propose and evaluate many generations of functions, they can be prohibitively computationally expensive. In contrast, dictionary learning algorithms approximate a function purely as a linear combination of candidate "dictionary" functions and can be efficiently fit using traditional linear regression methods.

The sparse identification of nonlinear dynamics (SINDy) algorithm [47] uses sparse dictionary learning to discover the governing equations for dynamical systems. We first present a general formulation of sparse dictionary learning and then make the connection to the different dynamics discovery formulations.

Dictionary Learning. Suppose we have collected N labeled data points $(\mathbf{x}_1, \mathbf{y}_1), \ldots (\mathbf{x}_N, \mathbf{y}_N)$, $\mathbf{x}_k \in \mathbb{R}^m$, $\mathbf{y}_k \in \mathbb{R}^n$ and would like to learn a function $\mathbf{y} = f(\mathbf{x})$ relating these variables. To create a dictionary model, we first compile a candidate set of d dictionary (or "library") functions (such as polynomials, sines, cosines, etc.):

$\mathbf{\Theta}(\mathbf{x}) = (\theta_1(\mathbf{x}), \theta_2(\mathbf{x}), \dots \theta_d(\mathbf{x}))$. We assemble data and label matrices, $\mathbf{X}$ and $\mathbf{Y}$ respectively

$$\mathbf{X} = [\mathbf{x}_1, \mathbf{x}_2, \dots, \mathbf{x}_N]^T \in \mathbb{R}^{N\times m}$$
$$\mathbf{Y} = [\mathbf{y}_1, \mathbf{y}_2, \dots, \mathbf{y}_N]^T \in \mathbb{R}^{N\times n},$$

and evaluate the dictionary on the collected data $\mathbf{X}$

$$\mathbf{\Theta}(\mathbf{X}) = [\theta_1(\mathbf{X}), \theta_2(\mathbf{X}), \dots, \theta_d(\mathbf{X})] \in \mathbb{R}^{N\times d}$$

to form the dictionary model:

$$\mathbf{Y} = \mathbf{\Theta}(\mathbf{X})\mathbf{\Xi} \tag{3}$$

where $\mathbf{\Xi} \in \mathbb{R}^{d\times n}$ are the coefficients to be fit. Sparse dictionary learning assumes that the desired function can be well approximated by a small subset of terms in the library, i.e. $\mathbf{\Xi}$ is a sparse matrix, as in the LASSO from statistics [101]. For dynamics discovery, where $\mathbf{y} = \dot{\mathbf{x}}$, this is physically motivated by the idea of parsimony—the governing equations for most physical systems have relatively few terms. To achieve this parsimony, a sparse optimization problem is formulated:

$$\mathbf{\Xi} = \underset{\hat{\mathbf{\Xi}}}{\operatorname{argmin}} \|\mathbf{Y} - \mathbf{\Theta}(\mathbf{X})\hat{\mathbf{\Xi}}\|_F^2 + \mathcal{R}(\hat{\mathbf{\Xi}}) \tag{4}$$

where $||\cdot||_F$ is the Frobenius norm and $\mathcal{R}(\mathbf{\Xi})$ is a sparsity-promoting regularization. . While the L^0 "counting norm" $\mathcal{R}(\mathbf{\Xi}) = \|\mathbf{\Xi}\|_0$ is the most appropriate to ensure sparsity, it is non-convex, non-continuous, and generally difficult to optimize when $d \gg 1$. For dynamics learning, sequentially thresholded least squares (STLS) has proven particularly effective for promoting sparsity by iteratively fitting the model and removing terms from the library whose coefficients are less than a certain magnitude [47, 51]. In our work, we consider a STLS with L^2 regularization (STLRidge) as in [68].

The SINDy algorithm is a particular case of the above framework when the data $\mathbf{X}$ are sampled points of the state space at a given time $\mathbf{x}_k = \mathbf{x}(t_k)$ and the labels $\mathbf{Y}$ are either the time derivatives $\dot{\mathbf{X}}$ or the next state $\mathbf{X}_{k+1}$ in the discrete setting; i.e. $\mathbf{y}_k = \dot{\mathbf{x}}(t_k)$ or $\mathbf{y}_k = \mathbf{x}(t_{k+1})$ respectively. These, and other dynamics discovery algorithms, are summarized with our notation in Supplementary Table 1: the SINDy-C algorithm [80] incorporates control by augmenting the state measurements with the corresponding control inputs, $\dot{\mathbf{X}} = \mathbf{\Theta}(\mathbf{X}, \mathbf{U})\mathbf{\Xi}$. Similarly, for discovering PDEs of the form $\psi_t = \mathcal{N}(\psi, \psi_x, \psi_{xx}, \dots)$, we can choose the data to be flattened snapshots of the solution field and any spatial derivatives, $\mathbf{X} = [\mathbf{\Psi}, \mathbf{\Psi}_x, \mathbf{\Psi}_{xx} \dots]$ and labels $\mathbf{Y} = \mathbf{\Psi}_t := \partial\mathbf{\Psi}/\partial t$.

Ensemble Learning. Ensemble-SINDy (E-SINDy) [77] introduced a way to build an ensemble of N_e SINDy models by bootstrapping data samples to create new datasets $\mathbf{X}^{(k)}, \dot{\mathbf{X}}^{(k)}$ and dictionaries $\mathbf{\Theta}^{(k)}$, $k = 1, 2, \dots N_e$ by dropping out some fraction of terms from the library. By choosing the median coefficients

$$\mathbf{\Xi}^*_{ij} = \text{Median}_k \left[\mathbf{\Xi}^{(k)}_{ij}\right] \tag{5}$$

the resulting SINDy model was more robust to noise, particularly in the low-data limit. In this work, we consider a generalization of the E-SINDy ensembling methods for arbitrary labeled datasets $\mathbf{X}, \mathbf{Y}$ by considering ensembles:

$$\mathbf{Y}^{(k)} = \mathbf{\Theta}^{(k)}(\mathbf{X}^{(k)})\mathbf{\Xi}^{(k)}$$

and taking the median coefficients $\mathbf{\Xi}^*$ as in Equation 5.

Algorithm	Discovery Task	$\mathbf{X}$	$\mathbf{Y}$
SINDy	$\dot{x} = f(x)$	$\mathbf{x}_k = \mathbf{x}(t_k)$	$\mathbf{y}_k = \dot{\mathbf{x}}(t_k)$
Discrete SINDy	$x_{n+1} = f(x_n)$	$\mathbf{x}_k = \mathbf{x}(t_k)$	$\mathbf{y}_k = \mathbf{x}(t_{k+1})$
Discrete SINDy-C	$x_{n+1} = f(x_n, u_n)$	$\mathbf{x}_k = (\mathbf{x}(t_k), \mathbf{u}(t_k))$	$\mathbf{y}_k = \mathbf{x}(t_{k+1})$
PDE-FIND	$\psi_t = \mathcal{N}(\psi, \psi_x, \ldots, x)$	$\mathbf{x}_k = (\psi, \psi_x, \ldots x)$	$\mathbf{y}_k = \psi_t(x, t_k)$

Supplementary Table 1: Different formulations of data-driven dynamics discovery with the sparse dictionary learning model $\mathbf{Y} = \mathbf{\Theta}(\mathbf{X})\mathbf{\Xi}$.

Uncertainty Quantification. The linearity of dictionary models not only provides a framework for interpretable models, but it also enables us to perform uncertainty quantification. For the scalar-valued dictionary model $y(\mathbf{x}) = \mathbf{\Theta}(\mathbf{x})\xi$ and $\xi \in \mathbb{R}^d$, it is simple to calculate the pointwise model variance under the assumption that the coefficients ξ are being drawn from some arbitrary distribution. Because $\mathbf{\Theta}(\mathbf{x})$ are independent from ξ, we can use the linearity of the dictionary model to write:

$$\mathrm{Var}_\xi[y(\mathbf{x})|\mathbf{x}] = \mathbf{\Theta}(\mathbf{x})\mathrm{Cov}(\xi)\mathbf{\Theta}(\mathbf{x})^T \tag{6}$$

where $\mathrm{Cov}(\xi)$ is the covariance matrix of the coefficients ξ. For a dictionary model of several variables $\mathbf{y}(\mathbf{x}) = (y_1(\mathbf{x}), \ldots y_n(\mathbf{x}))$ where $y_i(\mathbf{x}) = \mathbf{\Theta}(\mathbf{x})\xi_i$, the total variance of $\mathbf{y}(\mathbf{x})$ is given by the trace of its covariance matrix. Explicitly:

$$\mathrm{Tr}\left(\mathrm{Var}_{\mathbf{\Xi}}[\mathbf{y}(\mathbf{x})|\mathbf{x}]\right) = \sum_{i=1}^{n} \mathbf{\Theta}(\mathbf{x})\mathrm{Cov}(\xi_i)\mathbf{\Theta}(\mathbf{x})^T. \tag{7}$$

An ensemble of sparse models acts as an approximation of the coefficient distribution, and thus the pointwise variance can be rapidly computed just by estimating the covariance matrices of the fitted ensemble of coefficients.

Dictionary Learning for Control. By using SINDy-C [80], it is possible to exploit the dynamics for the purposes of control. Model predictive control (MPC) is a popular family of methods for optimal control that repeatedly optimizes control over a finite time horizon. This task can be incredibly expensive because it requires many forward evaluations of the dynamics in order to determine the optimal next step, and thus becomes challenging to deploy in real-time systems. In [81], SINDy was used to discover a lightweight dictionary model of the dynamics from limited data and used as a rapid surrogate model for MPC. This has been applied for the purposes of tokamak control [83]. SINDy has also been used to accelerate model-free DRL training using a surrogate SINDy model [86]; by leveraging random data to train a SINDy model, the authors show that it is sometimes possible to train a DRL agent without any additional data from the original environment.

There have also been approaches for online learning. By modeling unknown dynamics using SINDy and the unknown optimal value function as a quadratic form of the dictionary functions [84, 85], it is possible to use the Hamilton-Jacobi-Bellman equations to derive an expression for optimal control inputs. Then, by iteratively deploying control into the system, better dynamics models and value functions are learned. Finally, E-SINDy also explored the use of ensemble statistics for active learning: by identifying regions of the state space with high variance, the accuracy of the dynamics model can be efficiently improved by sampling trajectories from initial conditions from those regions.

	cartpole swing-up	Swimmer-v4	Cylinder	Pinball	3D Airfoil
**Environment**	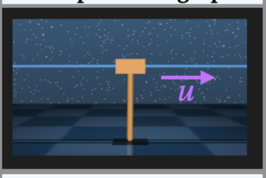	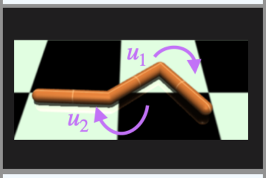	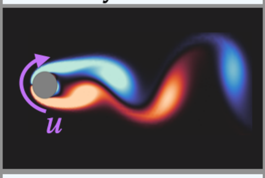	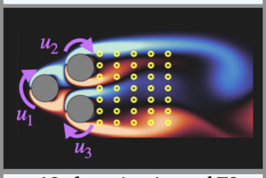	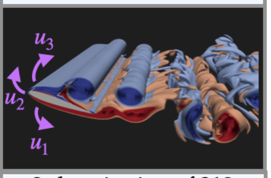
$\mathbf{x}_k$	$\left(x, \cos\theta, \sin\theta, \dot{x}, \dot{\theta}\right)$	$\left(\theta_1, \theta_2, \theta_3, v_x, v_y, \dot{\theta}_1, \dot{\theta}_2, \dot{\theta}_3\right)$	$(C_L,\ dC_L/dt)$	10-d projection of 70 velocity sensors	2-d projection of 318 velocity sensors
$\mathbf{u}_k$	Force in x-direction (1-d)	Torques to rotors (2-d)	Cylinder rotation (1-d)	Cylinder rotation (3-d)	Jet blowing / suction (3-d)
**Task**	Swing-up and stabilize pole	Maximize distance in x-direction	Minimize C_D	Minimize $C_{D1} + C_{D2} + C_{D3}$	Minimize $\lvert C_L - C_{L,ref}\rvert + \omega \lvert C_D - C_{D,ref}\rvert$

Supplementary Figure 2: The environments considered in this work, their respective observation spaces $\mathbf{x}_k$, and their action spaces $\mathbf{u}_k$. *Left:* The `swing-up` environment objective is to balance the pole on the cart in the upright (unstable) position starting from the downward (stable) position at rest by actuating the cart left and right. *Center Left:* The `Swimmer-v4` environment objective is to travel as far in the x-direction as possible in fixed time horizon starting from rest by actuating two rotors. *Center:* The Cylinder environment objective is to minimize the drag force on the cylinder surface by rotating the cylinder.
Center Right: The Pinball environment objective is to minimize the net drag force on the cylinder system by independently rotating the three cylinders. *Right:* The 3D Airfoil environment objective is to minimize fluctuations of the lift and drag forces from reference values.

3 Environments

As depicted in Supplementary Figure 2, we evaluate our methods among several different environments: the `dm_control` `swing-up` environment, the `gymnasium`[†] `Swimmer-v4` environment, the HydroGym Cylinder, the HydroGym Pinball environment, and the HydroGym-GPU 3D Airfoil environment. Below, we describe each environment in more detail.

3A Swing-up.

The `dm_control` `swing-up` environment uses continuous actions to swing up and balance a pendulum on a cart, starting from the downward position, by actuating the cart forward and backward along a horizontal rail. This is a more challenging controls task than the `gymnasium` "CartPole" (discrete actions) and "Inverted Pendulum" (continuous actions) benchmark environments which start near the upright positions—thus, `swing-up` mimics the more practical setup where the system starts in a stable, but undesirable configuration and requires sophisticated control to reach a region of interest (e.g. an unstable fixed point). As shown in Supplementary Figure 2, the states are given by the cart position x, pole angle θ, and their respective derivatives $\dot{x}, \dot{\theta}$, where the angle θ has been embedded on the circle S^1 to ensure continuous dynamics. The presence of the cart makes the dynamics significantly more complicated than a fixed pendulum. Without the cart, the pendulum dynamics can be recovered by dictionary learning with a library consisting of trigonometric and polynomial candidates. However, the presence of the cart leads to dynamics of the form $\mathbf{P}(\mathbf{x})\dot{\mathbf{x}} = \mathbf{Q}(\mathbf{x})$; thus $\dot{\mathbf{x}}$ cannot be easily expressed in terms of linear combination of elementary functions. While dynamics of this form have been addressed with dictionary learning in SINDy-PI [102] (by considering dictionary candidates for both $\mathbf{P}(\mathbf{x})$ and $\mathbf{Q}(\mathbf{x})$ and solving a more sophisticated optimization problem), we do not utilize that approach in this work. Instead, we demonstrate that even when the dynamics cannot be fully represented by our library, the imperfect representation is enough to achieve the controls task with SINDy-RL. As described in [103], the reward signal is given by a product of different functions bounded between 0 and

[†]formerly known as "OpenAI `gym`"

+1

$$r = \left(\frac{1+\cos\theta}{2}\right)\left(\frac{1+\Psi_1(x)}{2}\right)\left(\frac{4+\Psi_2(u)}{5}\right)\left(\frac{1+\Psi_3(\dot{\theta})}{2}\right),$$

where each Ψ_i are "tolerance" functions such that they are $+1$ near the desired behavior and decay to 0 away from it—providing a rich, but challenging learning signal. The combination of nonlinear dynamics/rewards, frequent interactions (100Hz), and long trajectory lengths (1000 interactions) makes this environment very challenging to explore.

3B Swimmer-v4

The `gymnasium` Mujoco environment `Swimmer-v4` consists of a 3-segment swimmer agent immersed in a viscous fluid as depicted in Supplementary Figure 2. The 8-dimensional observation space is comprised of the body orientation θ_1, the joint angles of the two rotors θ_2 and θ_3, their respective angular velocities ω_k, and the x- and y- velocities of the front segment's tip. By providing torque to the rotors on its two joints, the agent is tasked with moving as far as it can in the x-direction in a given number of time steps. To encourage this objective, the agent is rewarded with the body-centered x-velocity. However, this quantity is not provided as part of the agent's observation space. In order to use MBRL, a surrogate reward must be provided. It has been well-documented that—even with access to the exact rewards—solving the `Swimmer-v4` environment is extremely challenging because of the placement of the velocity sensors; researchers have even created their own modified versions of the environment when performing benchmarks [45]. It has been established across different benchmarks that the PPO algorithm struggles to achieve any meaningful performance despite its popularity and promising performance on other continuous controls tasks [104, 105]. Others have suggested that different hyper-parameters are needed [106].

3C Cylinder

The HydroGym Cylinder environment consists of the canonical two-dimensional unsteady fluid flow past a cylinder. This example is often used as a first approximation for many engineering systems that involve a fluid wake behind a blunt object, and it has become a popular test case for fluid flow control [26, 27, 107, 108]. By actuating the cylinder via rotations about its center, the flow around the surface of the cylinder changes and affects the forces it experiences. The objective is to minimize the drag, C_D, on the cylinder. The reward function is approximately $r = -\Delta t C_D$, where $\Delta t = 0.1$ is the amount of time between the agent taking actions. The dynamics of the flow are governed by the Navier-Stokes equations, and thus the full state of the system is infinite dimensional. To simulate this, HydroGym uses a finite element solver with thousands of degrees of freedom and, depending on the solver[‡] configuration, it may take several seconds to compute the effect of a single action, making it exceptionally time-consuming to use model-free RL. In many flow-control problems, engineers do not have access to all the desired sensing information. To emulate the under-observed setting in this work, we only provide the agent with observations of the lift coefficient and an estimate of its time derivative: $(C_L, \dot{C}_L)$; thus, in order to use MBRL, we must learn a representation of the reward function from our observations. From a theoretical perspective, these variables are motivated from the uncontrolled setting, where previous work has shown that flow can be described by measurements evolving on the $(C_L, \dot{C}_L)$ state space [56].

Because the C_L can be extremely sensitive to actuation, we first apply a low-pass filter

$$\hat{C}_L(t_{k+1}) = \hat{C}_L(t_k) + \frac{\Delta t}{\tau}(C_L(t_k) - \hat{C}_L(t_k)$$

[‡]In the version of HydroGym used to produce the Cylinder results, an incremental pressure correction scheme was used, causing step sizes to take longer. An updated version of the code uses a semi-implicit BDF solver, reducing the cost of a computational step.

where $\tau = 0.556 = T/10$, one-tenth of the vortex shedding frequency. We estimate $\dot{C}_L$ with an Euler estimate $\frac{\hat{C}_L(t_{k+1}) - \hat{C}_L(t_k)}{\Delta t}$. To achieve this, the solver timestep was set to $\Delta t = 0.01$ seconds with the agent interacting every 0.1 seconds (10Hz) and a simple first difference approximation was used.

Instead of simply setting the angular velocity of cylinder as performed in other fluid control simulators, the angular velocity of the cylinders are governed by the equation

$$\dot{\omega} = (\tau u - \omega)\frac{1}{\tau I_{CM}},$$

where u is the control input (torque), τ is the same time constant, and $I_{CM} = 0.0115846$ is the moment of inertia. For fixed control, the angular velocity will trend to τu. These dynamics act as a dampening caused by internal friction or external viscous dampening; thus providing a more realistic and complicated control scenario. The variable ω also acts as a hidden state variable since it is not given to the agent during training. In addition to the default actuation dampening in the environment provided every Δt, we provide a first-order hold in the intermediate timesteps between agent interactions in order to provide a smoother control signal into the environment.

For the initial off-policy data collection, we simulate the environment until the flow instability leads to periodic vortex shedding. Then we use 5 trajectories driven with sinusoidal control for 50 seconds followed by 10 seconds of no control. The k-th trajectory's sinusoid was parametrically defined by $u(t) = A\sin(\frac{2\pi t}{kT} - \phi) + B$, where $T = 5.56$ is the vortex shedding period of the single cylinder at Re=100—i.e. the period of each sine wave is an integer multiple of the shedding period. The parameters A, ϕ, B were sampled uniformly from the box: $[0.25, 1.0] \times [0, \pi] \times [-\frac{\pi}{2} + 1, \frac{\pi}{2} - 1]$ and chosen so that the maximal control of the environment $(\pm\pi/2)$ should never be exceeded.

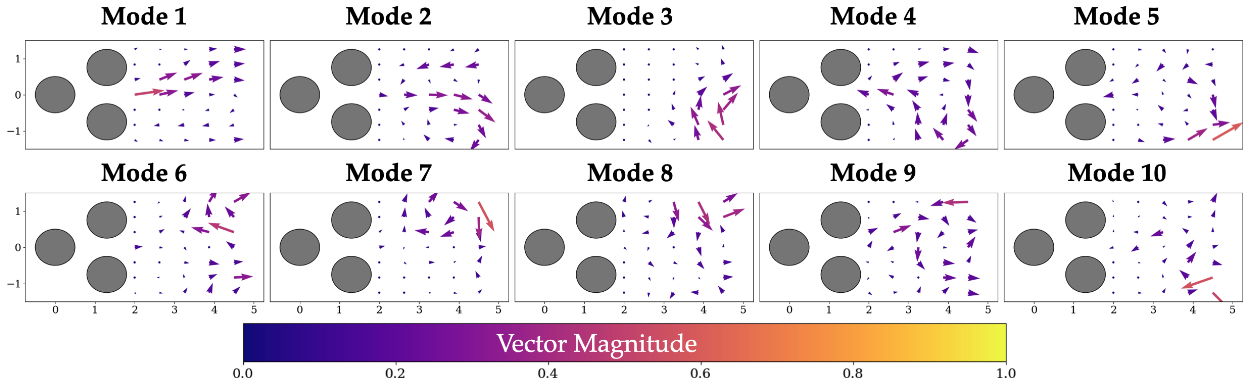

Supplementary Figure 3: Pinball SVD Modes. An illustration of the SVD modes obtained from snapshots at $Re = 100$ plotted as a vector-field at the sensor locations. Color indicates the magnitudes of the components at the given sensor.

3D Pinball

The "fluidic pinball" introduced Noack and Moryzński [109] consists of a 2-dimensional flow past three cylinders arranged in an equilateral triangle, as shown in Supplementary Figure 2. As the Reynolds number, Re, increases, the uncontrolled system dynamics experience a series of bifurcations. In particular, at $Re = 100$, the system exhibits quasi-periodic vortex shedding and at $Re \approx 115$, the dynamics become chaotic [110]. The objective of the environment is to minimize the total net drag $C_{D1} + C_{D2} + C_{D3}$ by independently rotating the three cylinders. In this version of the environment, observations are generated through a 7×5 grid of $x-$ and $y-$ velocity sensors in the wake as pictured in Supplementary Figure 2, for a total observation size of 70.

The initial condition was generated by solving for the steady state at $Re = 100$ and taking a sequence of 100 random actions to excite the instability in the flow and was simulated for an additional 200 seconds

without control with $dt = 0.01$ to reach the quasi-steady shedding. During this $200s$ period, snapshots of the observations were taken and used to obtain a low-dimensional SVD basis. To reduce the dimension of the observation, observations were projected onto the leading 10 SVD modes. Supplementary Figure 3 illustrates the modes as a vector field at the sensor locations. Many of the modes appear to be continuous vector fields highlighting vortex features in the wake.

Whereas the Cylinder was actuated at $dt = 0.1$, the Pinball is actuated at $dt = 0.01$—interacting with the environment 10 times as often. Just like the Cylinder, the actuation mechanism is applying torque to the cylinders until it saturates to some maximal angular velocity. In the version of HydroGym used for these results[§], though, the angular velocity is simplified to

$$\dot{\omega} = \tau(u - \omega)$$

where $\tau = 0.05$.

Just as the Cylinder, the initial off-policy data collection was produced from this uncontrolled initial condition and simulating forward with independently generated sinusoidal control on each of the three cylinders for 30 seconds followed by 5 seconds of no control (at $dt = 0.01$). The k-th trajectory's sinusoid was parametrically defined by $u(t) = A\sin(\frac{2\pi t}{kT} - \phi) + B$, where $T = 5.56$ is the vortex shedding period of the single cylinder at $Re = 100$—i.e. the period of each sine wave is an integer multiple of the shedding period. The parameters A, ϕ, B were sampled uniformly from the box: $[0.25, 0.75] \times [-\pi, \pi] \times [-0.25, 0.25]$ and chosen so that the maximal control of the environment ± 1 should never be exceeded.

3E 3D Airfoil

3E.1 Numerical Setup

The direct numerical simulations are conducted using a lattice Boltzmann method (LBM) with a cumulant-based collision operator implemented in the m-AIA (multiphysics - Aerodynamisches Institut Aachen) solver framework [111], which provides highly efficient and scalable computational capabilities. The framework benefits from hybrid parallelization based on MPI and shared memory models, enabling hardware-agnostic implementation on both CPU and GPU architectures with favorable strong and weak scaling on modern HPC systems [112]. The hierarchical unstructured Cartesian grids are generated using a massively parallel grid generator [113] integrated within m-AIA.

To enable efficient communication between the LB solver and reinforcement learning agents, m-AIA's MPI interface is extended using Multiple Program Multiple Data (MPMD) mode. This approach facilitates complex distributed computations across heterogeneous hardware while supporting different programming languages within the same application, enabling seamless integration between the CFD environment and Python-based RL libraries [112]. The MPMD interface enables frequent data exchange and coordination between different computational tasks with minimal computational overhead, supporting multi-agent and multi-environment training protocols in a straightforward fashion.

The LBM solver employs a D3Q27 velocity discretization scheme on hierarchical unstructured Cartesian grids. The lattice Boltzmann equation is formulated as:

$$f_i(\mathbf{x} + \boldsymbol{\xi}_i\delta t, t + \delta t) - f_i(\mathbf{x}, t) = -\omega(f_i(\mathbf{x}, t) - f_i^{eq}(\mathbf{x}, t)) \tag{8}$$

where f_i represents the particle probability distribution functions (PPDFs) at neighboring fluid cells, $\boldsymbol{\xi}_i = (\xi_{i1}, \xi_{i2}, \xi_{i3})^T$ denotes the discrete molecular velocity vector, and ω is the collision frequency [114]. The

[§]commit hash 0a19230a24a9b671893923c88a2da34899d79032

macroscopic flow quantities are obtained from moments of the distribution functions:

$$\rho(\mathbf{x}, t) = \sum_i f_i(\mathbf{x}, t) \tag{9}$$

$$\mathbf{u}(\mathbf{x}, t) = \frac{1}{\rho} \sum_i \boldsymbol{\xi}_i f_i(\mathbf{x}, t) \tag{10}$$

Following the cumulant approach of Geier et al. [115], instead of relaxing the momentum distribution function towards equilibrium in momentum space, countable cumulants are relaxed in cumulant space:

$$c_\alpha^* = c_\alpha + \omega_\alpha [c_\alpha^{eq} - c_\alpha] \tag{11}$$

where c_α^{eq} represents the Maxwellian equilibrium in cumulant space, and ω_α denotes the relaxation frequency. The cumulants are observable quantities that are Galilean invariant and statistically independent of each other [115]. Following standard practice, all relaxation rates except ω_1 are set to unity ($\omega_1 = \omega_{BGK}$, $\omega_\alpha = 1$ for $\alpha \neq 1$), where ω_{BGK} represents the BGK relaxation frequency.

The computational domain spans **[**$32\times$ **16** $\times 4$**]** chord lengths $c = 1.0$, with the NACA 0012 airfoil positioned **[8c]** from the inflow boundary. The domain extent is designed to minimize boundary reflections while maintaining computational efficiency. Non-reflecting characteristic boundary conditions are applied at the inflow and outflow boundaries to prevent pressure reflections [116]. The airfoil surface is treated with a no-slip boundary condition implemented through an interpolated bounce-back scheme. Periodic boundary conditions are imposed in the spanwise direction to simulate a nominally two-dimensional configuration while capturing three-dimensional flow instabilities. The domain height is set to 16 chord lengths to ensure far-field boundary effects do not influence the near-airfoil flow physics.

3E.2 Grid Resolution and Refinement Strategy

The simulations employ DNS where all relevant scales are directly resolved without the use of subgrid-scale models. The grid resolution is chosen to ensure that the smallest scales (Kolmogorov scales) are adequately captured, particularly in the separated flow regions downstream of the airfoil at $\alpha = 20°$. This approach provides the highest fidelity representation of the complex unsteady flow physics, including vortex shedding, flow separation, and turbulent mixing processes that are critical for accurate gust-airfoil interaction modeling.

The computational mesh employs an adaptive refinement approach with 14 refinement levels to efficiently resolve the disparate length scales present in the problem. Local grid refinement is implemented using hierarchical Cartesian grids with refinement patches positioned to capture critical flow regions. The spacing of the Cartesian grid on refinement level r is given by $\Delta x_r = L/2^r$, where Δx_0 represents the bounding box of the computational domain. The finest grid spacing near the airfoil surface is 1.953125×10^{-3} ensuring adequate resolution of the boundary layer and wake structures for DNS requirements. The numerical setup is validated against benchmark data from literature [117, 118] showing good agreement in time-averaged lift and drag coefficients across various angles of attack $0° < \alpha < 25°$ (see Fig. 4).

3E.3 Flow Conditions and Gust Implementation

The baseline flow conditions correspond to a chord-based Reynolds number of $Re_c = 1000$ at a Mach number of $M = 0.2$. The airfoil is positioned at a fixed angle of attack of $\alpha = 20°$, placing the system well into the post-stall regime where traditional linear aerodynamic models are invalid.

The transverse gust is implemented through a prescribed velocity perturbation imposed at the inflow boundary. The gust profile follows a 1-cosine approach with a gust factor $G = 2.0$, representing the ratio of

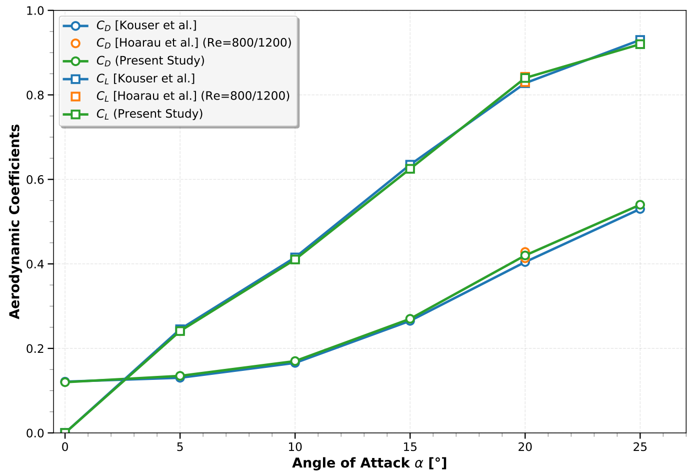

Supplementary Figure 4: Validation of numerical setup against benchmark data. Comparison of time-averaged lift (C_L) and drag (C_D) coefficients for NACA 0012 airfoil across various angles of attack ($0° < \alpha < 25°$) at $Re = 1000$. Present study results show good agreement with literature data (Kouser et al. [117] / Hoarau et al. [118]).

peak gust velocity to freestream velocity. The gust temporal development is controlled to achieve the desired interaction dynamics with the airfoil.

The control system leverages the HydroGym-GPU platform [112], which provides a standardized interface between high-fidelity CFD simulations and state-of-the-art reinforcement learning algorithms. This platform extends the original HydroGym framework by incorporating GPU-accelerated three-dimensional flow environments with the m-AIA solver, enabling efficient training of RL agents on complex fluid flow problems with grid sizes on the order of 10^8 cells.

Communication between the CFD environment and RL agents is facilitated through the MPMD interface, enabling real-time data exchange with minimal computational overhead [112]. This architecture supports multi-agent training protocols and allows for efficient exploration of the control parameter space during the learning process. All communications between environments and agents as well as inter-environment communication are handled by the MPMD interface, while relevant inter-agent communication is realized using existing deep learning libraries.

The airfoil-gust interaction environment is formulated as a discrete-time Markov Decision Process. Here, this discretization is inherent in the numerical simulation of fluid dynamics, which proceeds in discrete time steps. Starting from a given state (flow field), applying a control action results in a transition to a new state after a fixed interval of 640 time steps.

The state space encompasses 53 probes around the airfoil collecting horizontal and vertical velocity information across three different planes which are located at $z_p = [-1.0, 0.0, 1.0]$ (see Fig. 5). The collected probes values are normalized by the inflow velocity U_∞. To facilitate interactions between the RL agent and the flow environment, three jet actuators are distributed across the leading edge of the airfoil (see Fig. 5). Each actuator can be controlled independently, covers 3% of the chord length, and extends over the entire spanwise location. The action spaces are continuous and normalized in the range $[-1.0, 1.0]$. To simulate physical damping in the actuator and prevent numerical instabilities from high-frequency inputs, we model the actuators as leaky integrators.

The reward function is formulated to minimize gust-induced force fluctuations while maintaining aerodynamic efficiency:

$$R(t) = -|C_L(t) - \overline{C_{L,ref}}| - \omega\,|C_D(t) - \overline{C_{D,ref}}| \tag{12}$$

where $C_L(t)$ and $C_D(t)$ are the instantaneous drag and lift coefficients while $\overline{C_{L,ref}}$ and $\overline{C_{D,ref}}$ represent time-averaged coefficients of the unperturbed reference case. The weighting coefficient $\omega = 0.25$ is tuned to

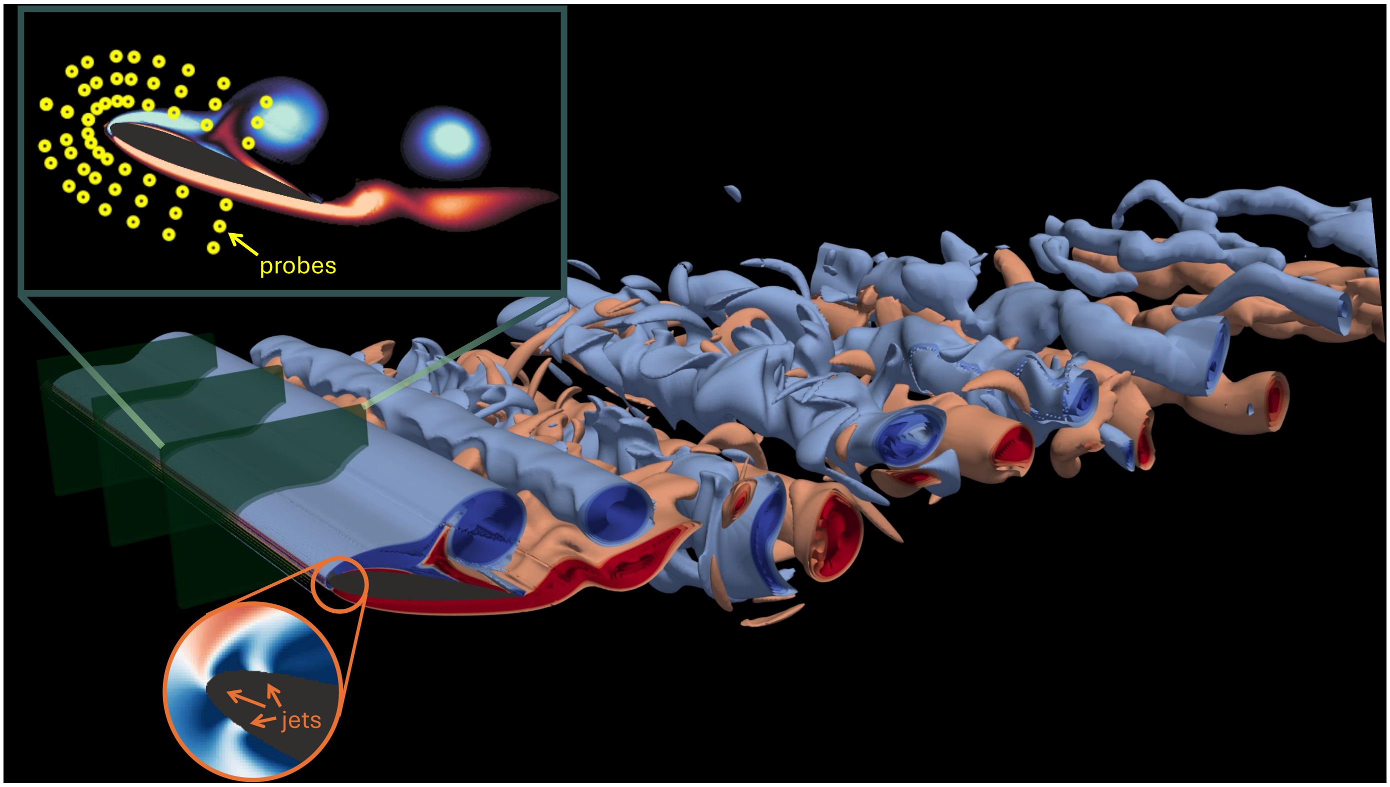

Supplementary Figure 5: Computational domain setup, sensor probe distribution and actuation strategy: Three-dimensional view showing the probe distribution around the NACA 0012 airfoil with vortical structures visualized through Q-criterion isosurfaces. The yellow dots in upper zoom-in highlight sensor probe locations in a single spanwise planes. Active flow control is performed using three independent jet actuators positioned along the leading edge of the NACA 0012 airfoil, each covering 3% of chord length and extending across the full span (see lower zoom-in).

balance the competing objectives of gust mitigation and drag reduction.

To compute the observations for both the SINDy-RL and baseline DRL experiments, the 318-dim measurements (= 2 velocities $\times$ 3 planes $\times$ 53 probes) were projected onto a 2-dim space. The space was obtained by collecting 10 episodes worth of data from random actuation of the flow. The mean sensor values were subtracted, and we project onto the leading 2 SVD modes from the resulting data matrix.

4 DRL Training and Benchmarking

Supplementary Table 2 provides the hyperparameters for each environment used in the Dyna-style SINDy-RL (Algorithm 1). We collect experience from the full-order environments in the form of a data store $\mathcal{D}$ consisting as a union of the off-policy data store $\mathcal{D}_{\text{off}}$ and on-policy data store $\mathcal{D}_{\text{on}}$. In an effort to not bias the dynamics too heavily towards the learned control policy, $\mathcal{D}_{\text{on}}$ employed a queue that removed old on-policy experience as new experience was collected. Other approaches to balance the two datasets, such as data augmentation or prioritized replay buffers [35] that strategically sample from the set of all collected data could also be employed, but were not considered in this work. To generate initial observations for the surrogate SINDy dynamics, we use the same sampling function for the full-order environment initial conditions. In the case of the HydroGym environments (where full-flow field checkpoints are needed to generate the initial conditions), we simply sample elements of $\mathcal{D}$ to initialize an episode of surrogate experience.

Environment	N_{collect}	N_{off}	n_{batch}
`swing-up`	1000	8000	40
`Swimmer-v4`	1000	12000	5
Cylinder	200	3000	25
Pinball	1000	17500	25
3D Airfoil	250	10000	25

Supplementary Table 2: Algorithm 1 Hyperparameters

For all of the DRL experiments involving the `swing-up`, `Swimmer-v4`, Cylinder, and Pinball, we utilize RLlib version 2.6.3. For the 3D Airfoil environment, we implement PPO directly in PyTorch to avoid issues with the HPC cluster and GPU m-AIA code.

RLlib. For each of the RLlib experiments, 20 different instantiations (e.g. random seed initializations) were used to characterize the variability of agents. Unless otherwise specified, we use RLlib's default parameters. For training the Baseline, Dyna-NN, and SINDy-RL algorithms, we use RLlib's [119] default implementation of PPO [120] and draw hyperparameters most similarly from CleanRL's exhaustive benchmarks [105]. All hyperparameters explicitly given to RLlib to override the default are provided in Supplementary Table 3. Just like CleanRL's benchmarks, we use annealing for the learning rate—decreasing linearly between an initial learning rate: 3.0×10^{-4} and 3.0×10^{-9}. Each neural network policy was a fully-connected neural network with two hidden layers consisting of 64 units each and hyperbolic tangent activation functions. For the Baseline PPO, Dyna-NN, and all SINDy-RL experiments (across all environments), policy updates are applied after 4000 interactions of experience from data collected in the (surrogate or full-order) environment.

γ	λ	VF Loss Coeff	PPO Clip	VF Clip	Gradient Clip
0.99	0.95	0.5	0.2	0.2	0.5

Supplementary Table 3: PPO Hyperparameters used in experiments.

Differences for 3D Airfoil For the 3D Airfoil, we use a custom implementation of PPO using PyTorch for both the baseline PPO and the policy algorithm $\mathcal{A}$ in Algorithm 1. Hyperparameters were chosen to match RLlib and those in Supplementary Table 3. Due to computational constraints, only 4 independent instantiations of a run were deployed for the 3D Airfoil in comparison to 20 instantiations for all other environments/algorithms. However, there was little performance variability between the agents.

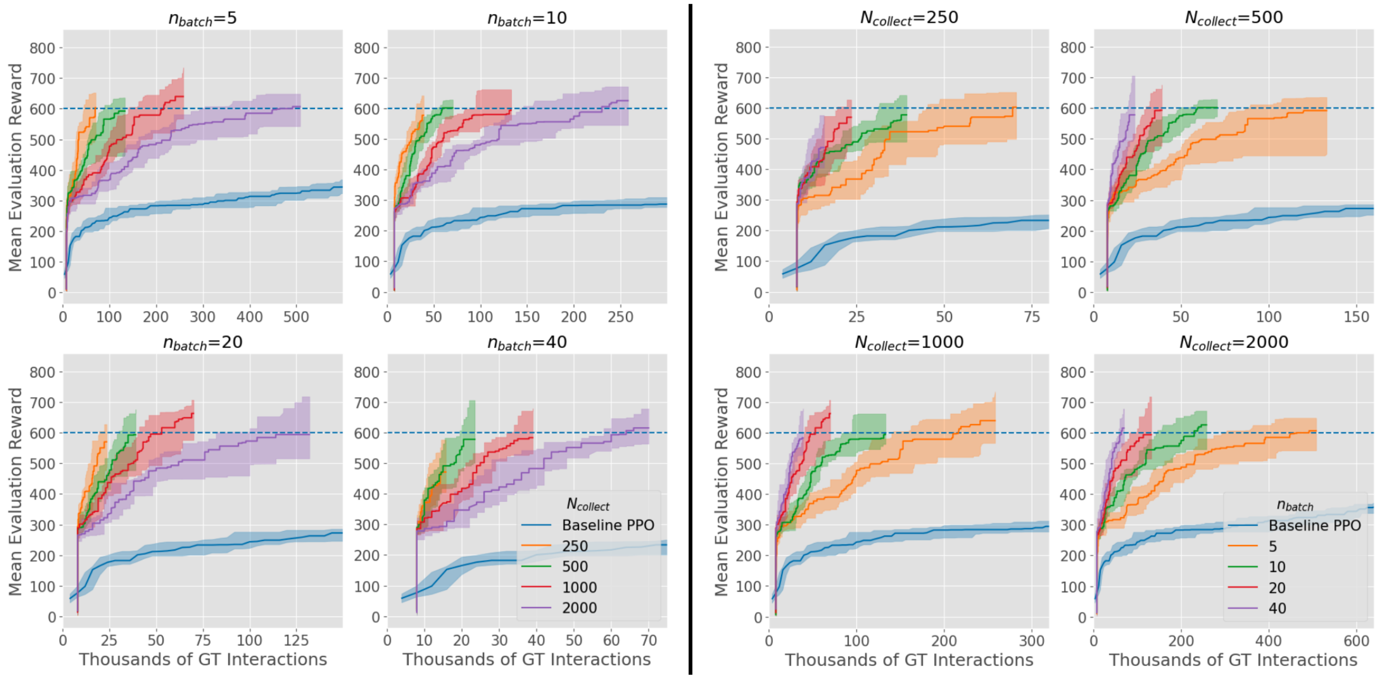

Supplementary Figure 6: A comparison of the number of policy updates, n_{batch}, and the number of on-policy experience, N_{collect}, collected from the `swing-up` environment before refitting the dynamics. The dashed blue line corresponds to the median best performance achieved in the baseline comparison. *Left*: Each plot compares $(n_{\text{batch}}, N_{\text{collect}})$ pairs where n_{batch} is fixed and N_{collect} varies. *Right*: Each plot compares $(n_{\text{batch}}, N_{\text{collect}})$ pairs where N_{collect} is fixed and n_{batch} varies.

4A MBRL Comparison—Swing-Up

For the SINDy-RL (linear and quadratic) and Dyna-NN experiments presented in the benchmark results, the cosine and sine components of the observation are projected back onto the unit circle after each interaction and the surrogate environments are reset if certain thresholds are exceeded (as described in Supplementary Section 5). For training the Dyna-NN dynamics, we use an ensemble of 5 neural network models (each a 2-hidden layer MLP with 64 hidden units per layer). The data is randomly split into an 80-20 training and validation split and trained for 50 epochs using PyTorch's Adam optimizer with a batch size of 500. Early stopping was used if the validation loss began to increase.

For the MB-MBPO comparison, we try to employ hyperparameters from the original paper (and RLlib implementation) for the `half-cheetah` environment because of its comparable state-dimensions. The MB-MPO policy is the same architecture, but with 32 nodes per hidden layer and trained with a fixed learning rate of size 3×10^{-4}. The MB-MPO dynamics were represented by an ensemble of 5 fully-connected neural network models with 2 hidden layers of size 512. Each dynamics model was trained for 500 epochs with early stopping if a running mean of the validation loss began to increase. For a fair comparison, the MB-MPO implementation collects 8000 interactions of random experience at the beginning of training and 1000 for every on-policy update.

On-Policy Collection Study. With SINDy-RL, we seek to minimize the number of interactions in the full-order environment. To this end, we investigate the effect that the number of policy updates per the dynamics update, n_{batch}, and the amount of on-policy data collected before each dynamics update, N_{collect}. We perform a grid search using $N_{\text{collect}} \in \{250, 500, 1000, 2000\}$ and $n_{\text{batch}} \in \{5, 10, 20, 40\}$ with the `swing-up` environment and train each environment for 1250 policy updates (5M samples of surrogate environment updates) using an initial off-policy collection of 8000 random steps and a maximum on-policy queue of equal size. Just as in

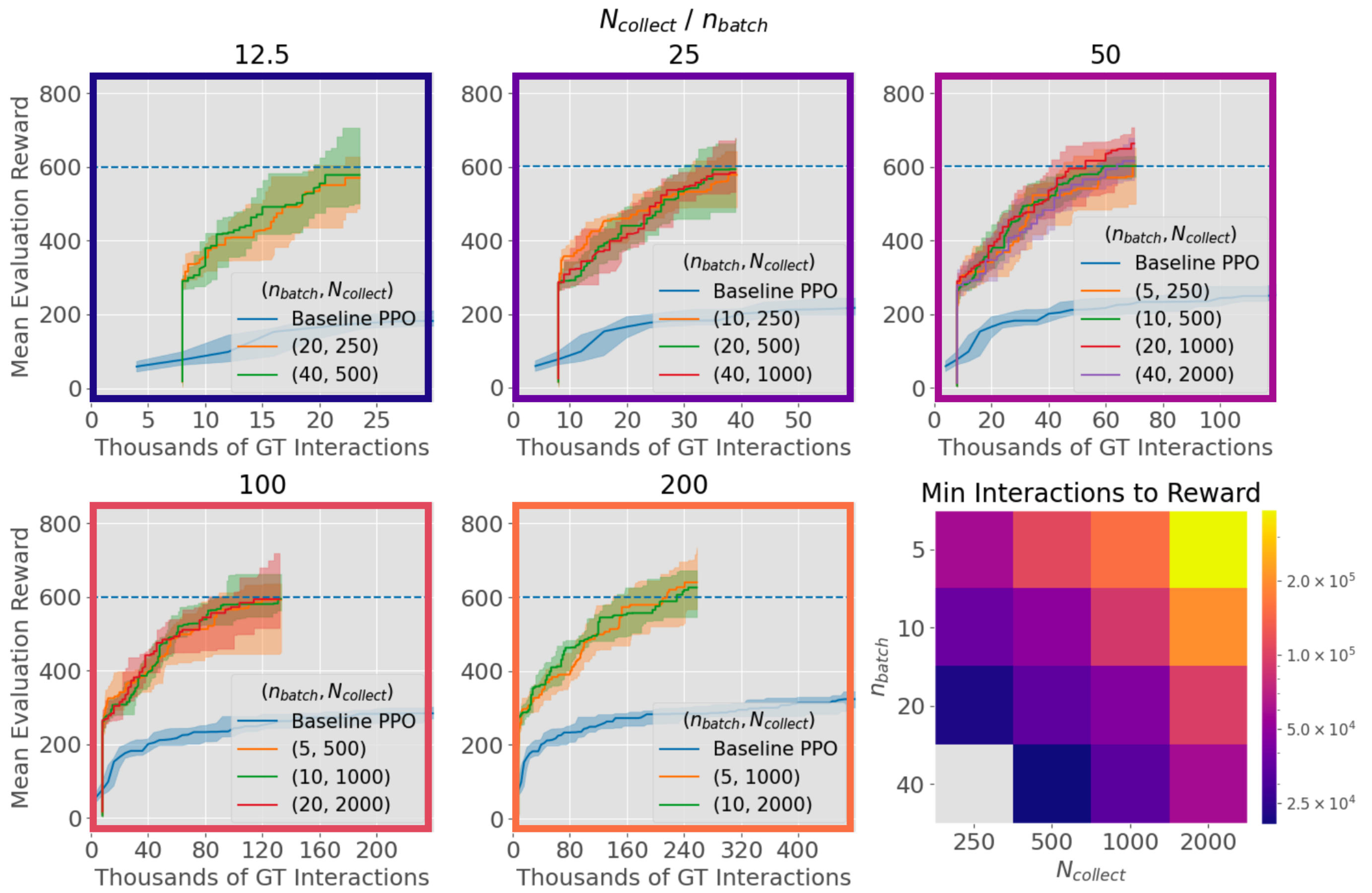

Supplementary Figure 7: A comparison of the number of policy updates, n_{batch}, and the number of on-policy experience, N_{collect}, collected from the `swing-up` environment before refitting the dynamics. The dashed blue line corresponds to the median best performance achieved in the baseline comparison. Each of the five timeseries plots are organized into combinations of $(n_{\text{batch}}, N_{\text{collect}})$ with fixed ratios. The bottom right heatmap indicates the minimal number of interactions for the median agent to have achieved a reward of 570. The diagonal level sets of the heatmap verify similar performance for the ratios. The (250, 40) combination remains empty because this combination's median best performance never reached the desired threshold.

the benchmarks from the main text, we evaluate performance on 20 different random seeds and track each policy's best performance. Supplementary Figure 6 demonstrates two different views of the data. Remarkably, the final performance is approximately the same for all $(n_{\text{batch}}, N_{\text{collect}})$ combinations, indicating that for the most part, selecting higher values of n_{batch} and lower values of N_{collect} will provide the most rapid policy improvement while minimizing the number of interactions in $\mathcal{E}$. However, the $(n_{\text{batch}}, N_{\text{collect}}) = (40, 250)$ combination does have the minimal performance, indicating that there is an optimal sampling frequency/size. In Supplementary Figure 7, we separate experiments by the ratio $N_{\text{collection}}/n_{\text{batch}}$. We clearly see that members from each ratio perform comparably for a fixed budget of $\mathcal{E}$ interactions, indicating that the ratio is a more important quantity than which combination results in the ratio. However, we believe that for extremely large values of n_{batch}, the neural network will overfit to the imperfect dynamics much faster than if it fit more frequently with smaller samples.

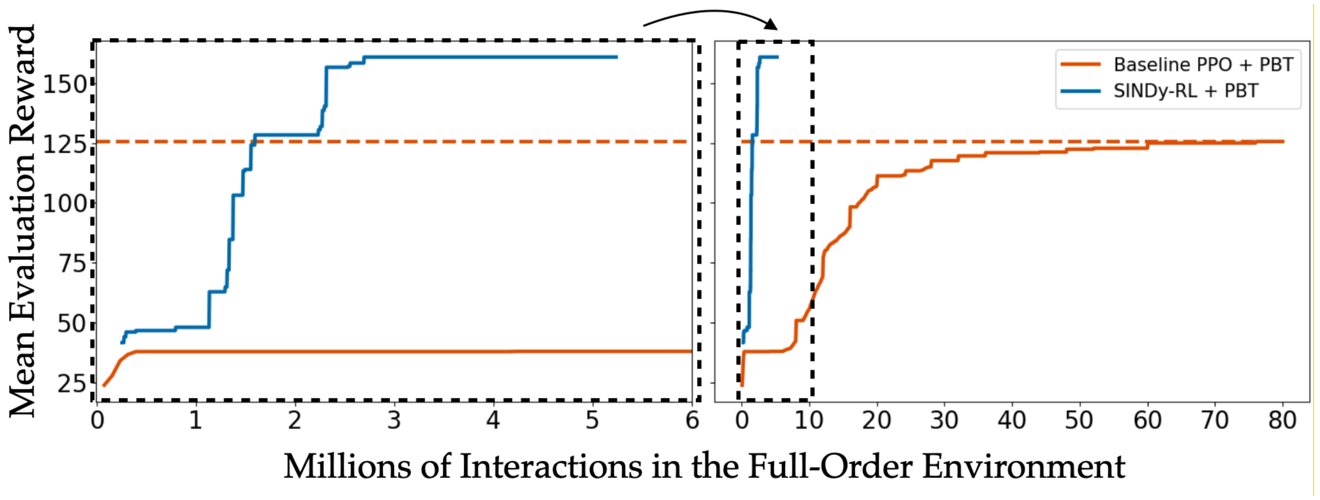

Supplementary Figure 8: Swimmer Sample Efficiency. Comparison of the total number of interactions in the full-order `Swimmer-v4` environment using population-based training (PBT) for PPO hyper-parameter tuning using a population of 20 experiment seeds. Each experiment consists of a single policy and is periodically evaluated over 5 episodes. The population's best evaluation reward is tracked, recorded, and updated. The dashed red line shows the baseline PPO+PBT best final reward.

4B Population-Based Training—Swimmer-v4

In population-based training (PBT), a population of candidate policies are trained in parallel using different random seeds and DRL hyperparameters. Periodically the members of the population are evaluated: the bottom performers are stopped and resampled from the top performers with some corresponding mutation (such as changing a hyperparameter). PBT and other population-based frameworks have become a popular method of hyperparameter tuning and avoiding local equilibria that neural networks are extremely prone to. However, PBT for DRL struggles from a practical perspective because these methods must evaluate—not just one, but—many policies in the full-order environment.

We employ the Ray Tune [121] and RLlib implementation for PBT to tune PPO hyperparameters. Evaluation and resampling occur synchronously every 50 policy updates (200k experience collected from either the surrogate environment for the SINDy-RL implementation or the full-order environment for the baseline PPO). For each experiment, we use a population size of 20 different policies. When evaluating and resampling, the performers from the bottom quartile are stopped and restarted using the policies from the upper quartile. New hyperparameters are either chosen from the original distribution at random or seeded and mutated from the top performer that was copied. Although each hyperparameter can be drawn from a continuous range, we only consider a discrete set of combinations. Explicitly, we consider the following hyperparameter combinations:

$$\begin{aligned}\text{LR} &\in [1\times10^{-6}, 5\times10^{-6}, 1\times10^{-5}, 5\times10^{-5}, 1\times10^{-4}, 5\times10^{-4}, 1\times10^{-3}, 5\times10^{-3}]\\ \lambda &\in [0.9, 0.95, 0.99, 0.999, 0.9999, 1.0]\\ \gamma &\in [0.9, 0.95, 0.99, 0.999, 0.9999, 1.0]\end{aligned}$$

all other hyperparameters are fixed and the same as used in the other PPO experiments. The results can be seen in Supplementary Figure 8.

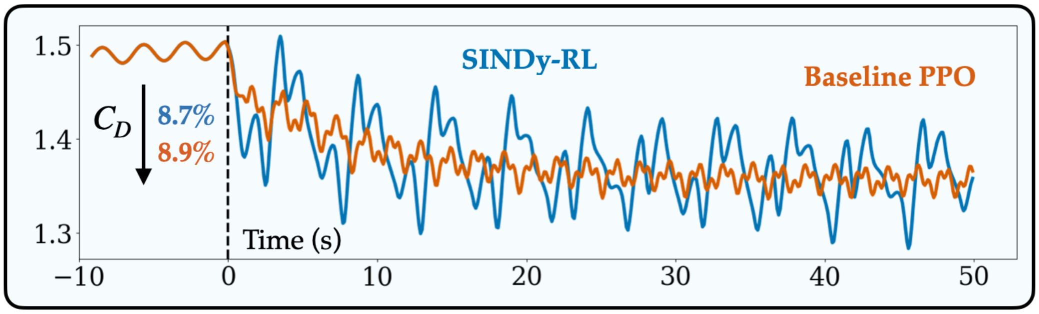

Supplementary Figure 9: Cylinder Medium Mesh Comparison. Evaluation of the best performing SINDy-RL and baseline agents using the fine mesh after 4,600 and 100,000 full-order interactions respectively. Control starts at $t = 0$ marked by the black dashed line. For ease of visualization, the 1-second window-averaged C_D value is plotted for both agents.

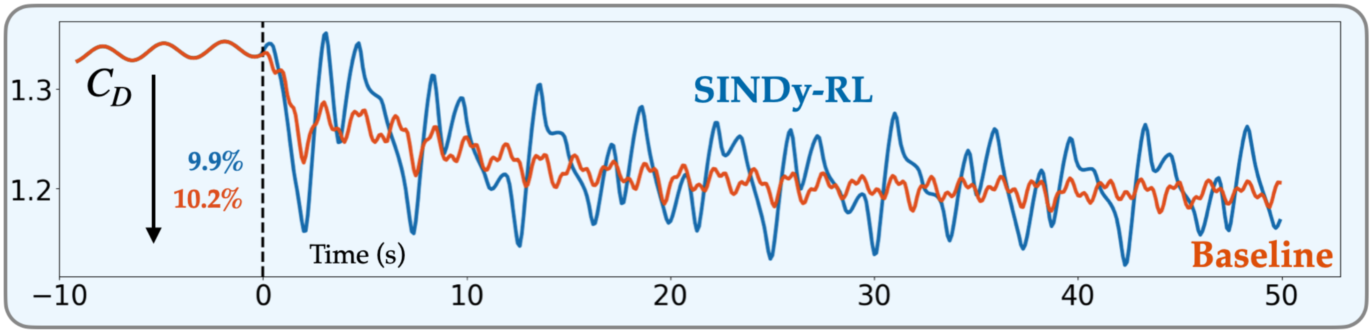

Supplementary Figure 10: Cylinder Fine Mesh Comparison. Evaluation of the best performing SINDy-RL and baseline agents using the fine mesh after 4,600 and 100,000 full-order interactions respectively. Control starts at $t = 0$ marked by the black dashed line. For ease of visualization, the 1-second window-averaged C_D value is plotted for both agents.

4C Cylinder Benchmark

HydroGym also supports a fine mesh consisting of hundreds of thousands of finite elements. Because of its massive size, it's not recommended for training on the Cylinder. However, our agents only receive information about the lift coefficients and are thus mesh-agnostic. While we never collect data from the fine mesh, we can still deploy our trained agents to it. Figures 9 and 10 depict the agents from the benchmarks in the main text being applied to the medium and fine mesh, validating that our approach translates to unseen, higher-fidelity environments.

4D Pinball Benchmark

In Supplementary Figure 11, we plot the comparison of the net drag experienced on the system with and without control for the SINDy-RL and baseline approaches for various Re when only being trained at $Re = 100$. For each Re, the two approaches provide similar values for net drag with the SINDy-RL agent oscillating near zero, and veering more into the negative (higher rewards) corresponding to larger thrust production.

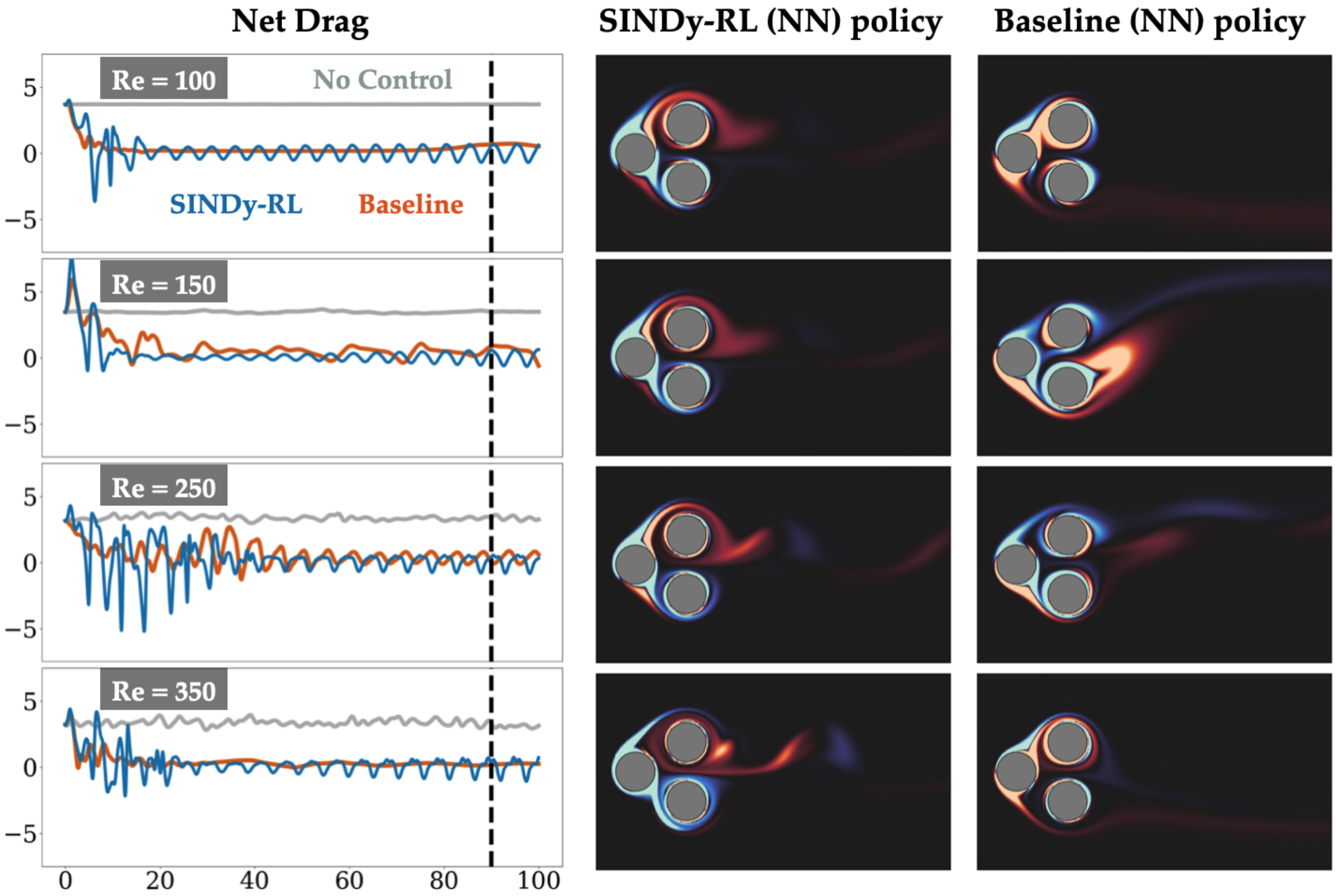

Supplementary Figure 11: Pinball Re evaluation. Evaluation of the best performing SINDy-RL and baseline agents across Re. *Left:* Net drag experienced on the system for the SINDy-RL agents (blue), baseline agents(red), and without control (gray). Negative values indicate thrust generation. Dashed line at $t = 90$ corresponds to the snapshots on the right. *Right:* Vorticity snapshots at $t = 90$.

Env	Full step	Surrogate step	Surrogate policy training	Evaluation	E-SINDy Fit
Cylinder	6.86s	1.55×10^{-3}s	3.46×10^{2}s	$\mathbf{1.37 \times 10^{3}}$s	5.9×10^{-2}s
Pinball	1.78s	3.77×10^{-3}s	4.59×10^{2}s	$\mathbf{1.56 \times 10^{3}}$s	13.5s
3D Airfoil	45s	3.49×10^{-4}s	3.20×10^{2}s	$\mathbf{1.13 \times 10^{3}}$s	5.0×10^{-2}s

Supplementary Table 4: Fluid Environment clock-times. A comparison of different clock-times for SINDy-RL on the HydroGym environments. "Full step" and "Surrogate step" measure the amount of time used to take a single step in the full-order and surrogate environments—for the Cylinder and Pinball, this was derived from Ray-RLlib "mean_env_wait_ms" metric; for the 3D Airfoil all values were estimated manually from training logs. "Surrogate policy training" refers to the amount of time used to train the policy in the surrogate environment before updating the surrogate models (for 25 policy updates with 4000 interactions with the surrogate per step). "Evaluation" refers to the amount of time for evaluating the environment (i.e. collecting N_{collect} worth of experience and updating the surrogate environments). "E-SINDy Fit" refers to the amount of time to fit the 20-member ensemble of E-SINDy models in serial using the *maximal* amount of data allowed by the datastore buffer.
Note: the actuation frequency used for the Cylinder environment was 10 times smaller than the Pinball (10Hz compared to 100Hz), resulting in a much larger simulation time in between actions.

4E 3D Airfoil Benchmark

In Supplementary Figure 12 we compare the effect of the SINDy-RL policies and the baseline PPO policy. Both agents are able to drive the C_L force closer to the desired reference value much sooner than the uncontrolled case, with the SINDy-RL agent performing slightly better. The SINDy-RL agent also does a better job of mitigating the gust for the C_D value, but to a lesser extent; however, the PPO baseline agent actually increases the amount of drag on the airfoil.

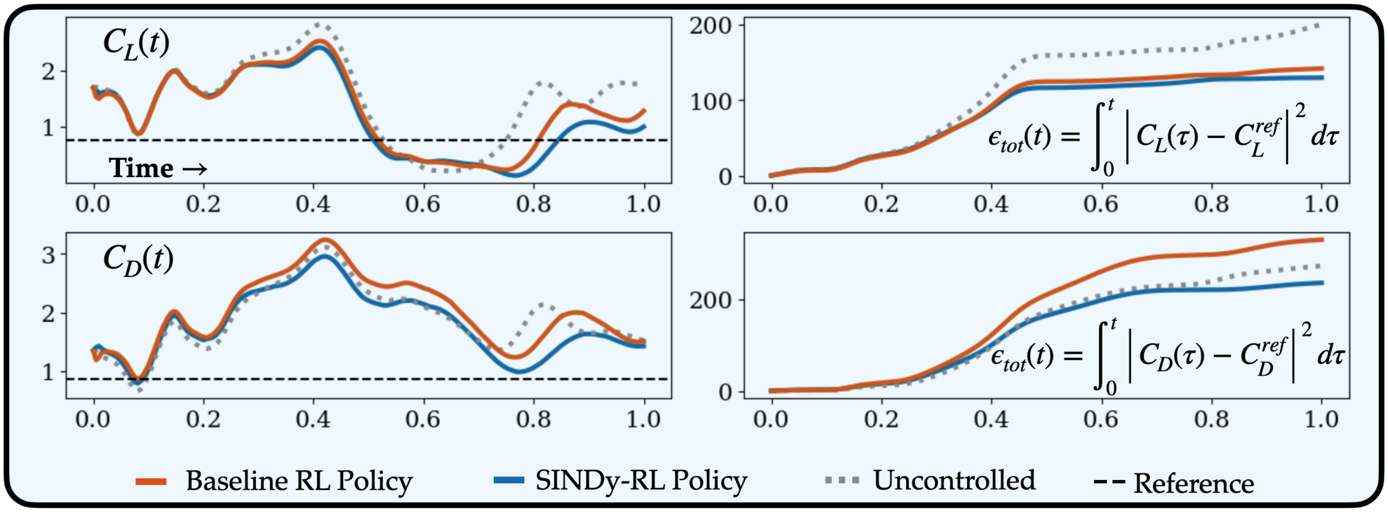

Supplementary Figure 12: 3D Airfoil Evaluation. Evaluation of the best performing SINDy-RL and baseline agents. *Left:* Net lift and drag experienced on the system for the SINDy-RL agents (blue), baseline agents(red), and without control (gray dotted). *Right:* Cumulative error from the desired reference values.

5 Dictionary Dynamics

We learn the surrogate dictionary dynamics and reward functions for all environments, with the exception of the `swing-up` task where we only learn the dynamics. For the results presented in this work, we restrict ourselves to learning discrete control-affine dynamics

$$x_{n+1} = f(x_n) + g(x_n)u_n \tag{13}$$

where f and g are polynomials of some maximal degree, though a much broader class of libraries and SINDy-algorithms can be used, such as SINDy-PI [102]. For dictionary learning, we use `PySINDy`'s [122, 123] E-SINDy implementation with an ensemble of $N_e = 20$ models using STLRidge. Supplementary Table 5 summarizes the libraries and hyperparameters for each model. During the Dyna-style training in Algorithm 1 from the main text, trajectories are terminated and the surrogate environments are reset if the observations exceed environment specific thresholds, which are detailed below.

Environment	$f(x)$	$g(x)$	Thresh	α	Ensemble
`swing-up`	Quadratic	Quadratic	7×10^{-3}	5×10^{-5}	Median
`Swimmer-v4`	Quadratic (no cross)	Quadratic (no cross)	2×10^{-2}	5×10^{-1}	Median
Cylinder	Cubic	1	1×10^{-3}	1×10^{-5}	Median
Pinball	Quadratic	1	1×10^{-3}	1×10^{-5}	Median
3D Airfoil	Quadratic (no cross)	Quadratic (no cross)	1×10^{-2}	1×10^{-5}	Median

Supplementary Table 5: For each environment, the library types for f, g (Eq. 13) are listed with the sparsity threshold and ridge regression weight, α. "Ensemble" refers to whether the median or mean coefficients were used. No f libraries include a bias term. "no cross" implies no cross terms in the quadratics.

5A Swing-up

As shown in Supplementary Figure 14, we use the affine library as in Equation 13 where f and g are polynomials of maximal degree 2. Note that because our state-space includes $\sin(\theta)$ and $\cos(\theta)$ terms, there are redundant combinations of library functions due to the trigonometric identity: $\cos(\theta)^2 + \sin(\theta)^2 = 1$. To accommodate for this redundancy, we do not incorporate a constant offset into the library. The final learned dynamics are given by the equation below, highlighting the Euler-like update, which gives rise to the diagonal entries in Supplementary Figure 14.

$$\begin{aligned}
x_{k+1} &= 1.000x_k + 0.010\dot{x}_k \\
\cos(\theta_{k+1}) &= 1.000\cos(\theta_k) - 0.010\sin(\theta_k)\dot{\theta}_k \\
\sin(\theta_{k+1}) &= 0.999\sin(\theta_k) + 0.010\cos(\theta_k)\dot{\theta}_k \\
\dot{x}_{k+1} &= 0.998\dot{x}_k + 0.063u_k + 0.032\cos(\theta_k)^2 u_k + 0.030\sin(\theta_k)^2 u_k \\
\dot{\theta}_{k+1} &= 1.000\dot{\theta}_k + 0.148\sin(\theta_k) + 0.005x_k u_k \\
&\quad - 0.142\cos(\theta_k)u_k - 0.007x_k^2 u_k + 0.004x_k\cos(\theta_k)u_k
\end{aligned}$$

To fit the SINDy model, we first collect $N_{\text{off}} = 8000$ interactions (80 seconds) of random experience to fit the dynamics. For rolling out the surrogate dynamics, we assume prior knowledge that the angle is embedded on the circle $(\cos(\theta), \sin(\theta))$ and renormalize after every step taken in the surrogate dynamics. During training, we collect $N_{\text{collect}} = 1000$ interactions (10 seconds) worth of on-policy experience for every dynamics update, and $\mathcal{D}_{\text{on}}$ employed a queue of size 8000.

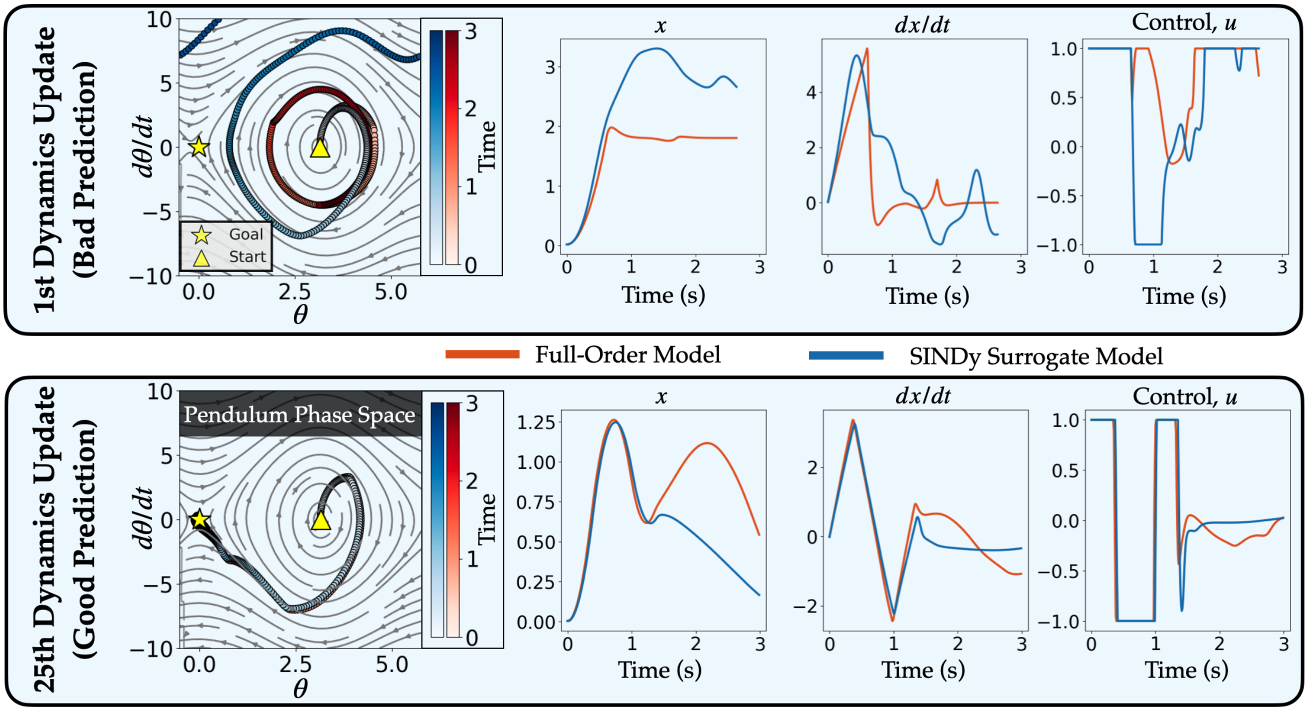

Supplementary Figure 13: Swing-up Dynamics. The learned dynamics and control of the `dm_control swing-up` environment by rolling out the DRL control policy in the high fidelity and surrogate environments after the 1st and 25th dynamics updates. *Left:* The phase portrait for the angle, θ (evaluated at $x = \dot{x} = u = 0$ for ease of visualization), and the corresponding DRL-agent trajectories of the full-order and surrogate models. *Right:* The DRL-agent trajectories of the full-order and surrogate models for the $x, \dot{x}$ components of the state and the corresponding control.

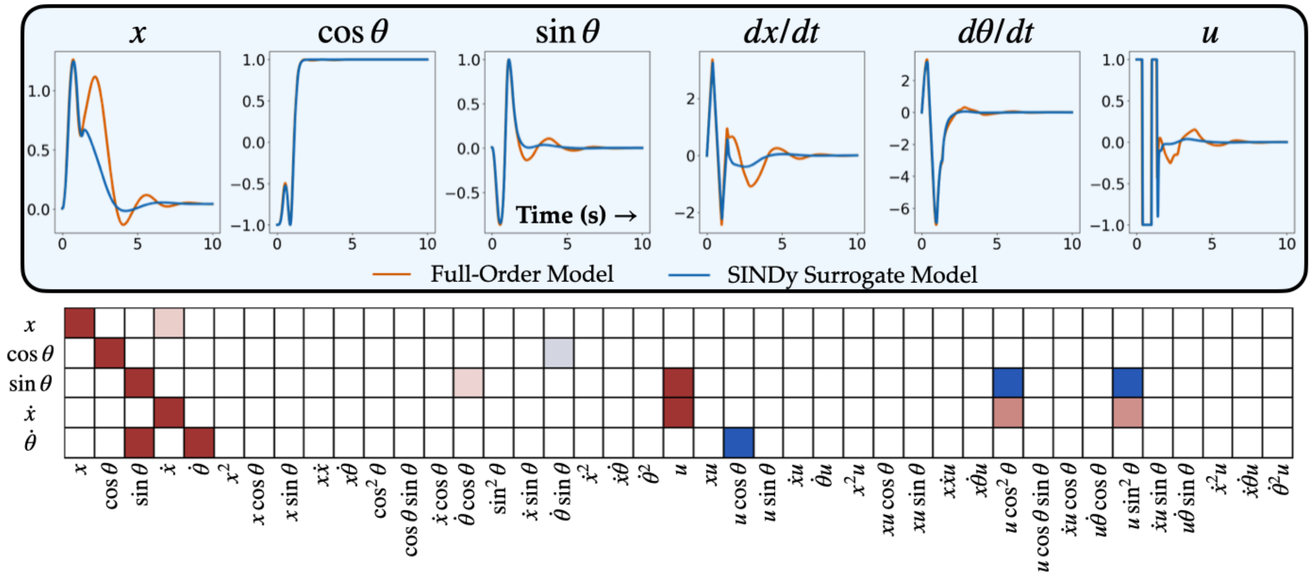

Supplementary Figure 14: Swing-Up Dynamics. *Top:* Rollouts of the SINDy surrogate (blue) and full-order (red) dynamics under the influence of feedback control of the learned neural network policy in each respective environment after being trained with Algorithm 1. *Bottom:* A heatmap of the learned dictionary coefficients, Ξ. For ease of visualizing smaller coefficients, each row's coefficients are normalized by the maximum value and the color map clips between small positive (red) and negative (blue) value of equal magnitude to demonstrate the sign.

A trajectory is terminated and the surrogate environment is reset if the observations exceed the following

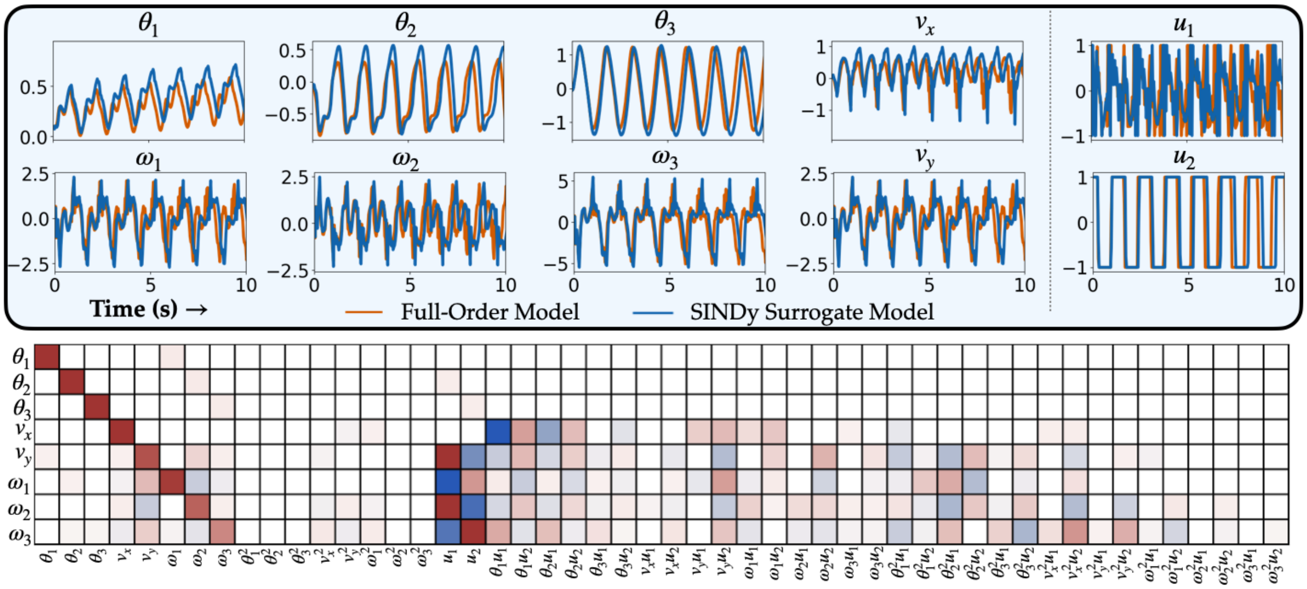

Supplementary Figure 15: Swimmer-v4 Dynamics. *Top:* Rollouts of the SINDy surrogate (blue) and full-order (red) dynamics under the influence of feedback control of the learned neural network policy in each respective environment after being trained with Algorithm 1. *Bottom:* A heatmap of the learned dictionary coefficients, Ξ. For ease of visualizing smaller coefficients, each row's coefficients are normalized by the maximum value and the color map clips between small positive (red) and negative (blue) value of equal magnitude to demonstrate the sign.

thresholds:

$$|x| \leq 5, \qquad |\cos\theta| \leq 1.1, \qquad |\sin\theta| \leq 1.1, \qquad |\dot{x}| \leq 10, \qquad |\dot{\theta}| \leq 10$$

For the linear dynamics models used in the benchmark comparison, we use a linear-affine library (with bias) and set the threshold and regularization coefficients to zero.

5B Swimmer-v4

Just as in the `swing-up` example, we use `PySINDy` to fit the dynamics (along with the reward function—see Supplementary 6 for more details). As shown in Supplementary Figure 15, we use a quadratic dynamics library in terms of the observation components without the interacting terms; the learned dynamics match well with the full-order environment under the influence of the control policy. Unlike the `swing-up` environment, we make no assumptions on the manifold that the state-space live when rolling out the surrogate dynamics. However, we do include bounds:

$$|\theta_1| \leq \pi, \qquad |\theta_2|, |\theta_3| \leq 1.7453, \qquad |v_x|, |v_y|, |\dot{\theta}_k| \leq 10$$

We bound θ_2 and θ_3 so that we do not reach the maximal angle for the swimmer robot. Physically, it is reasonable to assume knowledge of this ahead of time in order to not damage the physical asset. This also avoids the piecewise dynamics needed to describe the environment. If there is prior knowledge about how the dynamics behave near the boundary these could also be incorporated (e.g. clipping the state and zero-ing out the velocity terms) but was not explored in this work.

5C Cylinder

As shown in Supplementary Figure 16, the Cylinder dynamics were fit using a 3rd degree polynomial (without interaction terms) in the state variables $(C_L, \dot{C}_L)$ and a completely isolated control term. A conservative set of bounds were used on the observation space:

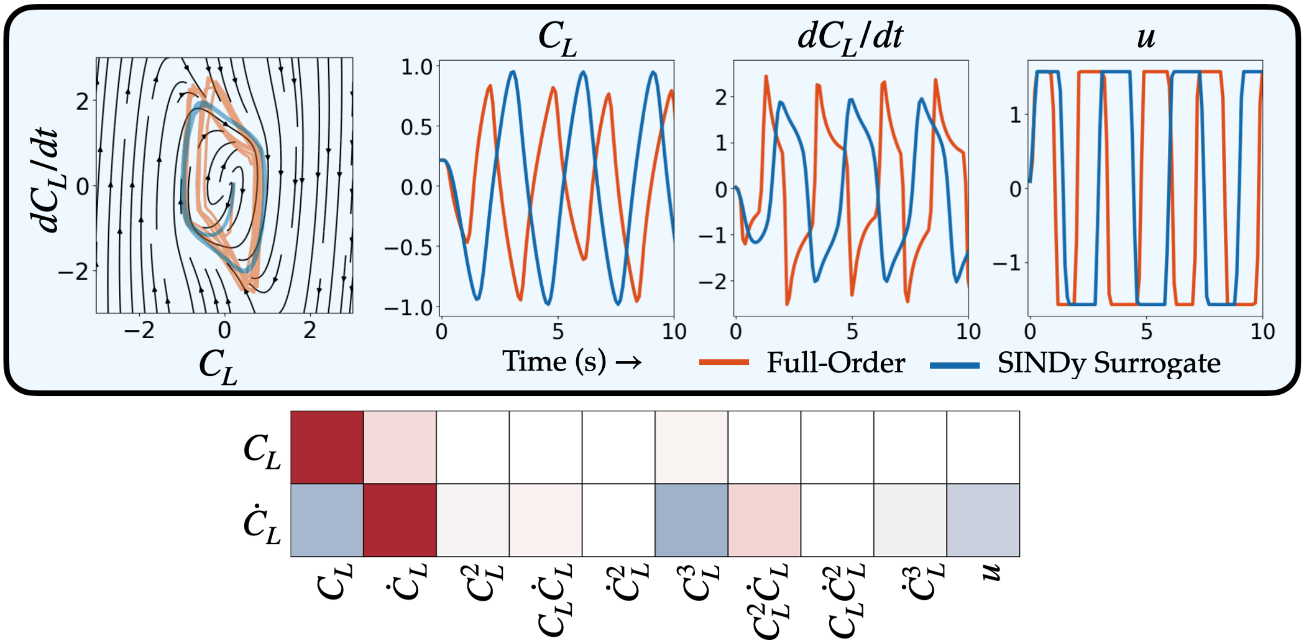

Supplementary Figure 16: Cylinder Dynamics. *Top:* Rollouts of the SINDy surrogate (blue) and full-order (red) dynamics under the influence of feedback control of the learned neural network policy in each respective environment after being trained with Algorithm Algorithm 1. A vector field of the learned dynamics with feedback control is plotted in phase space on the left. *Bottom:* Heatmap of the learned dictionary coefficients, Ξ. For ease of visualizing smaller coefficients, each row's coefficients are normalized by the maximum value and the color map clips between small positive (red) and negative (blue) value of equal magnitude to demonstrate the sign.

$$|C_L| \leq 10, \qquad |\dot{C}_L| \leq 200$$

Under the influence of feedback control, a stable limit cycle emerges in the surrogate dynamics. However, as indicated by the data plotted in red, the true dynamics vary slightly and leave the cycle.

5D Pinball

As shown in Supplementary Figure 17, the Pinball dynamics were fit using a 2nd degree polynomial in the SVD coefficients a_i and completely isolated control terms, u_i. A conservative set of bounds were used on the observation space:

$$|a_i| \leq 20$$

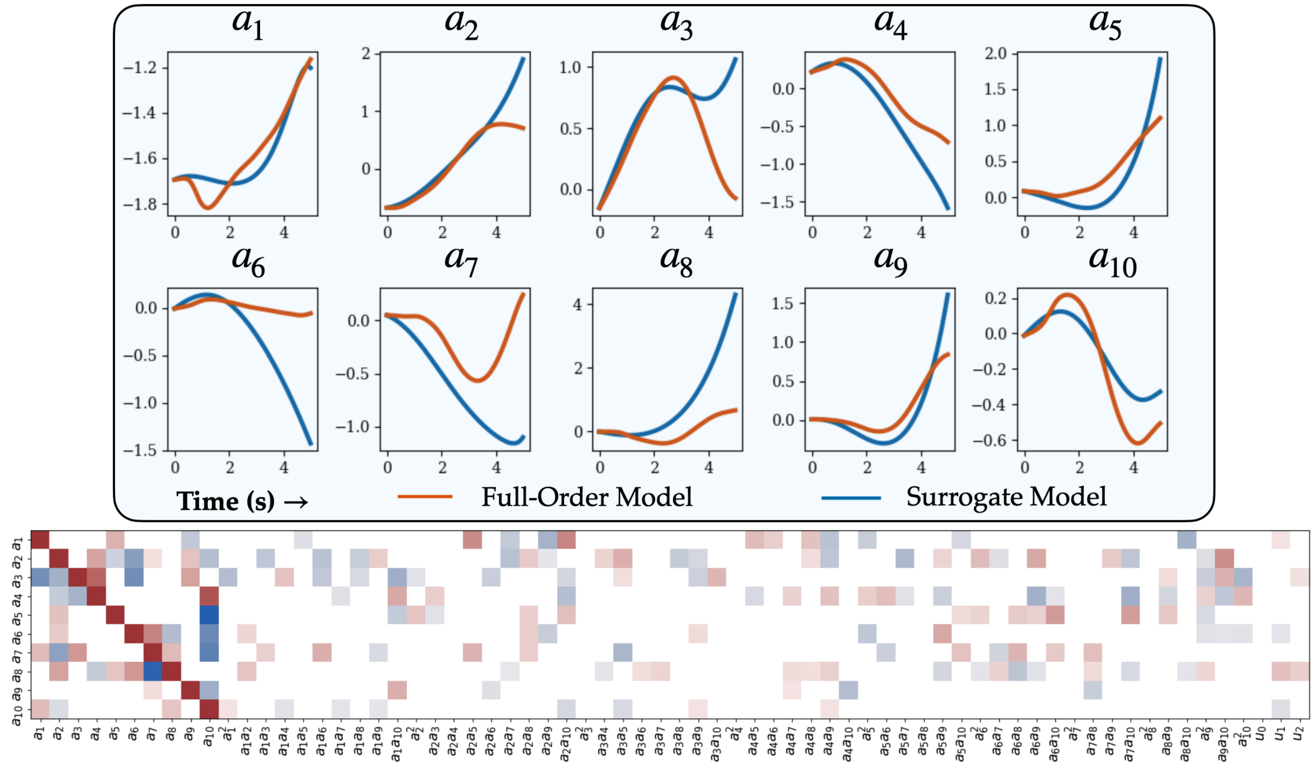

Supplementary Figure 17: Pinball Dynamics. *Top:* Rollouts of the SINDy surrogate (blue) and full-order (red) dynamics under the influence of feedback control of the learned neural network policy in each respective environment after being trained with Algorithm 1. *Bottom:* Heatmap of the learned dictionary coefficients, Ξ. For ease of visualizing smaller coefficients, each row's coefficients are normalized by the maximum value and the color map clips between small positive (red) and negative (blue) value of equal magnitude to demonstrate the sign.

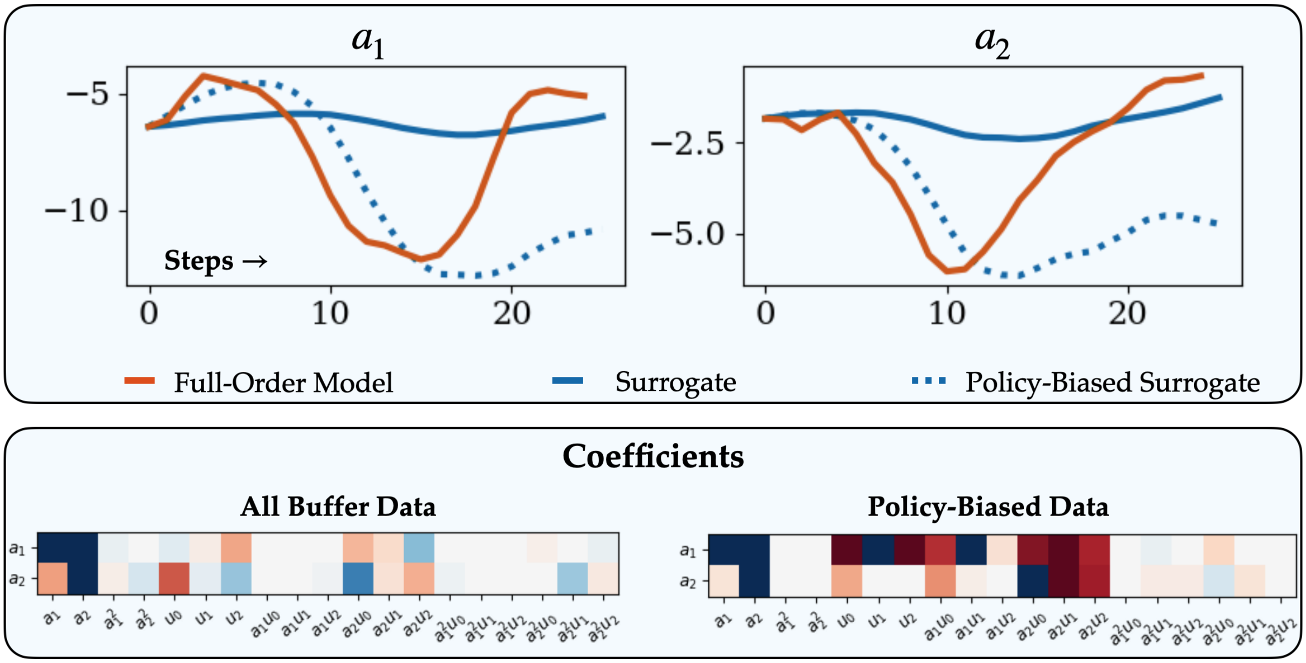

Supplementary Figure 18: 3D Airfoil Dynamics. *Top:* Rollouts of the SINDy surrogate (blue) and full-order (red) dynamics under the influence of feedback control of the learned neural network policy in each respective environment after being trained with Algorithm 1. Solid blue line corresponds to the learned dynamics model during training that used all 10 of the initially collected trajectories and all subsequent evaluations. Dotted blue line corresponds to a biased model that was only trained on 3 of the collected data and all subsequent evaluations (Note: biased model is only included as a demonstration; it was not used during training). *Bottom:* Heatmap of the learned dictionary coefficients, Ξ for the learned dynamics model during training (left) and the model that was biased on the data collected from training the policy (right). For ease of visualizing smaller coefficients, each row's coefficients are normalized by the maximum value and the color map clips between small positive (red) and negative (blue) value of equal magnitude to demonstrate the sign.

5E 3D Airfoil

As shown in Supplementary Figure 18, the 3D Airfoil dynamics were fit using a 2nd degree polynomial in the SVD coefficients a_i and control terms u_i. A conservative set of bounds were used on the observation space:

$$|a_i| \leq 1000$$

For training our SINDy-RL agents, used the *entirety* of the off-policy buffer (consisting of the initial random trajectories) was used and was sufficient to train a meaningful control policy. However if the dynamics for the randomly controlled agent are substantially different than the controlled agent, then it may be favorable to prune those trajectories later during training. Supplementary Figure 18 demonstrates the difference in rolling out the final surrogate dynamics model that used all the available data vs. a model that only used 30% of the initial random trajectories (i.e. one that is biased towards the on-policy collected data). While the biased-dynamics struggle to forecast the final behavior of the trajectory, the dynamics agree reasonably well for the first half of the trajectory. In contrast, when using the full dataset, the influence of control is greatly tempered. This can be verified by examining the coefficients; the biased model exhibit a much stronger dependence on the control terms.

6 Dictionary Rewards

Just as with the dynamics, we use `PySINDy`'s E-SINDy implementation with $N_e = 20$ models and STLRidge. All models use the median coefficients for predicting rewards. A summary of libraries and hyperparameters can be found in Supplementary Table 6.

Environment	Library	Thresh	α
`Swimmer-v4`	Quadratic*	5×10^{-2}	5×10^{-5}
Cylinder	Quadratic	1×10^{-4}	5×10^{-5}
Pinball	Quadratic	5×10^{-1}	1×10^{-5}
3D Airfoil	Cubic *	6×10^{-4}	1×10^{-5}

Supplementary Table 6: For each environment, the library types for the reward function are listed along with the STLS threshold and the ridge regression weight, α. * : The swimmer and 3D Airfoil libraries did not contain cross terms. The swimmer did not contain a bias, nor terms involving the control variables.

6A Swing-up

We do not learn the dictionary rewards as we have access to an analytic equation, as described in Supplementary 3. We also expect that it would be challenging to learn such a reward function because it is a product of four functions, each with an approximately quadratic Taylor Series—indicating that the rewards would only be well described near the equilibrium by an 8th order polynomial and would have trouble decaying away from the equilibrium. While a library with better properties could be examined, it was not investigated in this work.

6B Swimmer-v4

As illustrated in Supplementary Figure 19, we use a quadratic dictionary on the observation variables with no interaction terms to model the `Swimmer-v4` surrogate reward. Using median coefficients, the final learned reward was:

$$\hat{r} = 0.917 v_x - 0.086 \theta_1^2 + 0.141 v_x^2,$$

indicating that the x-velocity of the leading segment v_x is a good proxy for the body-center velocity that is not present. Supplementary Figure 19 depicts the predictions of the reward during a full-rollout of the controlled full-order environment, and indeed the rewards are well-correlated—especially for the cumulative return used to estimate the value function.

6C Cylinder

As illustrated in Supplementary Figure 20, a quadratic library with mixing terms was used to fit the surrogate reward function. The resulting model was found to be

$$\begin{aligned}\hat{r} = -(1.52 \times 10^{-1}) \quad &- (8.21 \times 10^{-4}) C_L \quad -(9.85 \times 10^{-4}) \dot{C}_L + (6.22 \times 10^{-3}) C_L \dot{C}_L \quad -(2.72 \times 10^{-3}) C_L^2 \\ -(5.94 \times 10^{-4}) u &+ (3.34 \times 10^{-3}) C_L u \quad -(1.34 \times 10^{-2}) \dot{C}_L^2 - (2.05 \times 10^{-2}) \dot{C}_L u \quad -(5.06 \times 10^{-3}) u^2\end{aligned}$$

In Supplementary Figure 20, the *raw* surrogate reward function is shown to be more sensitive than the full-order environment rewards, and does not provide very accurate immediate predictions. Despite this, the reward provided a suggestive enough learning signal to train SINDy-RL agents to successfully reduce the drag.

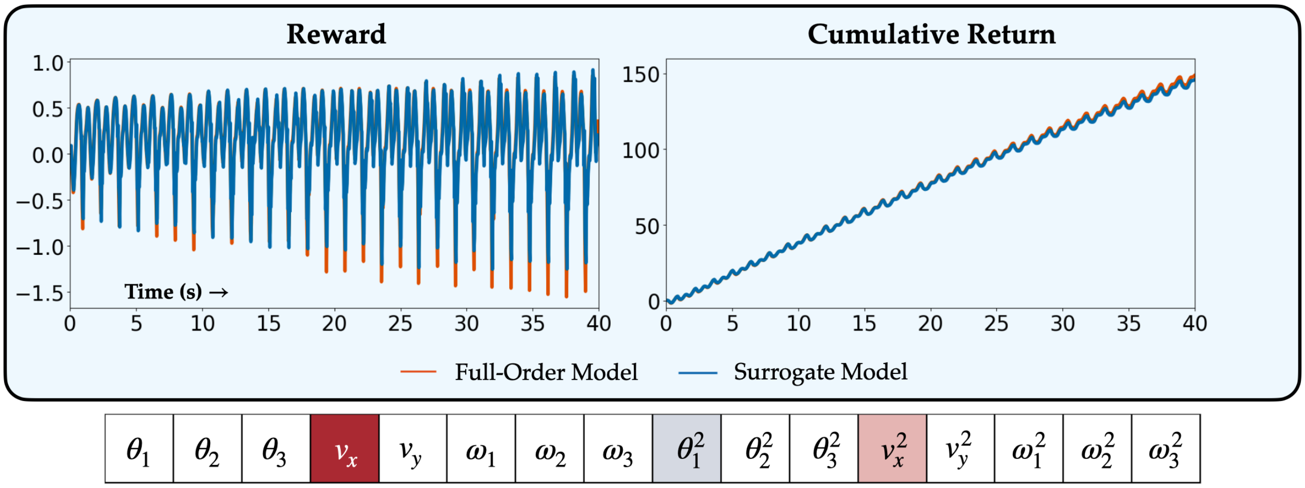

Supplementary Figure 19: Swimmer-v4 Reward. *Top:* Surrogate dictionary reward predictions evaluated on trajectories produced from the full-order environment. *Bottom:* A heatmap of the learned dictionary coefficients, Ξ. For ease of visualizing smaller coefficients, the row's coefficients are normalized by the maximum value and the color map clips between small positive (red) and negative (blue) value of equal magnitude to demonstrate the sign.

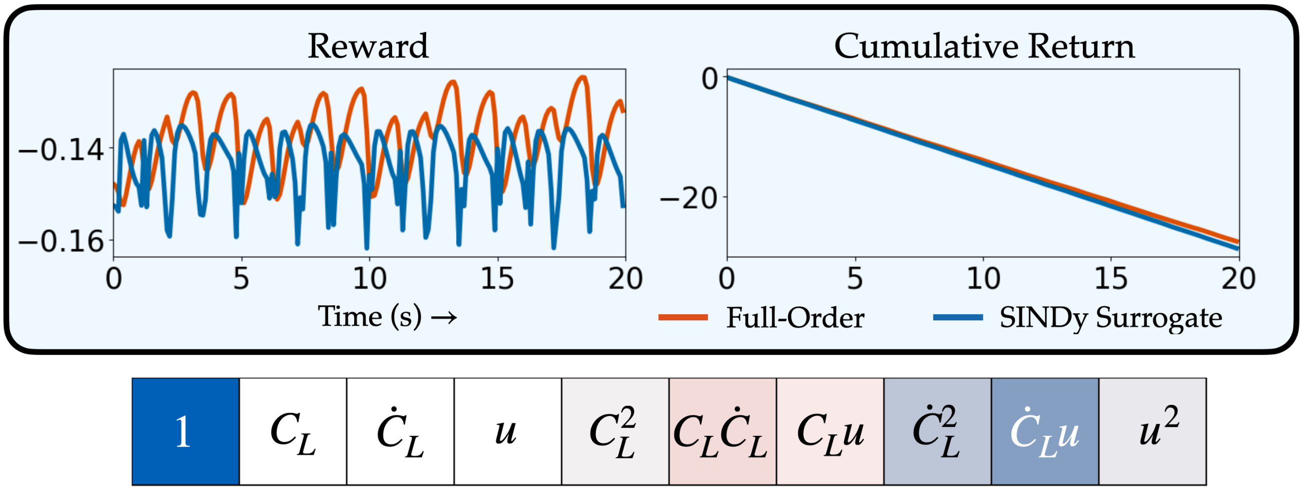

Supplementary Figure 20: Cylinder Reward. *Top:* Surrogate dictionary reward predictions evaluated on trajectories produced from the full-order environment. *Bottom:* A heatmap of the learned dictionary coefficients, Ξ. For ease of visualizing smaller coefficients, the row's coefficients are normalized by the maximum value and the color map clips between small positive (red) and negative (blue) value of equal magnitude to demonstrate the sign.

6D Pinball

In Supplementary Figure 11, the median surrogate reward function is shown to be more "optimistic" than the ground truth. While reasonably correlated, the median coefficients often predicts higher rewards, resulting in a net cumulative reward that is significantly larger. The individual members of the ensemble, however, have magnitudes closer to the ground truth. While not explored, this indicates that the *mean* coefficients might be better suited for the purposes of training, whereas the median are better for interpretability and compression. Through the sparsity promoting optimization, only 24 out of the 105 median coefficients are nonzero. Because the observation only contains information from sensors in the wake, the reward relies significantly on the control input, which directly affects the local field and fluid forces on the system. The result includes non-trivial coupling between the sensor modes and the actuation.

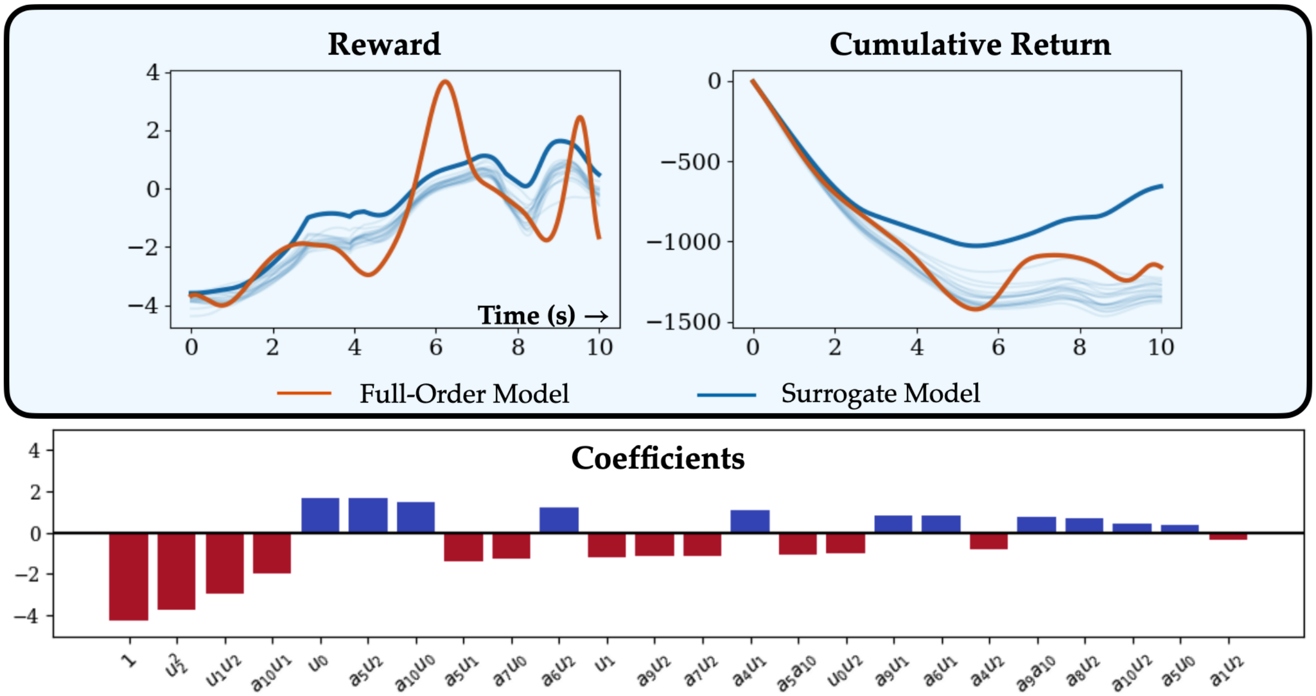

Supplementary Figure 21: Pinball Reward. *Top:* Surrogate dictionary reward predictions evaluated on a trajectory produced from the full-order environment. The blue bold line indicates the predictions with the surrogate model with *median* coefficients. The faint blue lines are individual predictions from members of the ensemble. *Bottom:* A comparison of the learned, non-zero dictionary coefficients, $\boldsymbol{\Xi}$, ordered by magnitude.

6E 3D Airfoil

The final airfoil reward was fit as a cubic polynomial of the projection onto the SVD modes a_i and the control inputs u_i (with no cross-terms forming the interaction). It's clear that the control terms dominate the learned reward.

In Supplementary Figure 22, we evaluate the (median) E-SINDy reward function on data from the final evaluation episode. The reward matches closely for most of the episode, but underpredicts the final values. Despite this, the model was still able to learn a competitive policy to the DRL benchmark.

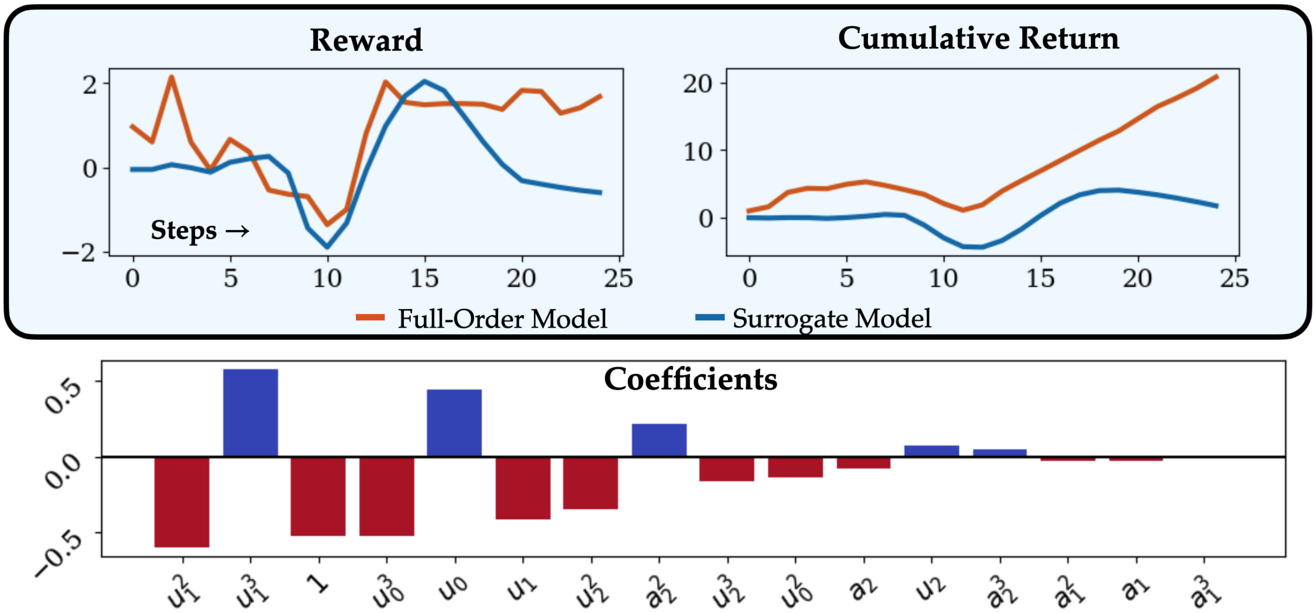

Supplementary Figure 22: 3D Airfoil Reward. *Top:* Surrogate dictionary reward predictions evaluated on a trajectory produced from the full-order environment. *Bottom:* A comparison of the learned, non-zero dictionary coefficients, Ξ, ordered by magnitude.

Env	Initial Conditions	Traj. Length	Noise	N_{dyn}
`swing-up`	$\theta \sim \mathcal{N}(0, 0.1), x = \dot{x} = \dot{\theta} \sim N(0, 0.25)$	500	0.1	5,000
`Swimmer-v4`	$x_i \sim N(0, 0.1)$	200	0.1	10,000
Cylinder	$x \sim \mathcal{D}_{\text{on}} + N(0, 0.1)$	10	0.1	10,000
Pinball	$x \sim \mathcal{D}_{\text{on}} + N(0, 0.1)$	100	0.1	10,000
3D Airfoil	$x \sim \mathcal{D}_{\text{on}} + N(0, 0.2)$	5	0.2	13,800

Supplementary Table 7: Summary of the parameters uses to fit each surrogate policy. "Initial condition" refers to how the initial conditions were chosen. "Traj. Length" is the maximal length of the surrogate trajectories. "Noise" refers to the scale of the zero-mean added Gaussian noise after collecting the trajectories. N_{dyn} refers to the total number of points collected using the surrogate dynamics.

7 Dictionary Policy

We fit the surrogate policy by compiling input observations, $\mathbf{X}$, evaluating them at points $\mathbf{Y} = \mathbb{E}\left[\pi_\phi(\mathbf{X})\right]$ and fitting an ensemble of cubic polynomial dictionary models $\hat{\pi}^{(k)}(\mathbf{x}) = \mathbf{\Theta}^{(k)}(\mathbf{x})\mathbf{\Xi}^k$ for $k = 1, 2, \ldots N_e = 20$. Below, in Supplementary 7A we discuss different sampling strategies for collecting the data $\mathbf{X}$. For all of the results presented in this work, we acquire initial conditions and use our accompanying surrogate learned dynamics models with feedback control from the neural network model to bootstrap new cheap experience without needing to access the full-order model. Just as in training Algorithm 1, we reset the surrogate environment if our trajectories begin to diverge. We apply a small amount of noise to the trajectories by sampling twice from a Gaussian distribution before querying the neural network policy. Finally, we sweep over the STLRidge hyper-parameters on a test set. Supplementary Table 7 summarizes the parameters for each of the environments. Note that while rolling out the policy in the real and surrogate environments, we clip the controls to be between a minimal and maximal value. However, to learn the dictionary policies, it is sometimes more effective to loosen this bound when creating the data $\mathbf{Y}$ to allow for smoother predictions. In all of our experiments, we clip between -5 and 5. While one could use completely unclipped values, it can risk adding unnecessary complexity to the resulting polynomial. Although sparsity was encouraged during the STLRidge sweep, the policies did not end up being truly sparse, and we found the mean coefficients to provide a more robust prediction than the median.

7A Policy Sampling Study

For the `swing-up` task, we consider six different ways of collecting the data for querying the neural network policy:

- Sampling from an ambient mesh in $\mathbb{R}^m$ based off the collected data.
- Projecting the ambient space onto a manifold with known constraints (i.e. the circle S^1)
- Sampling random trajectories from our learned dynamics and controller.
- Injecting noise onto sampled trajectories from the learned dynamics and controller.
- Sampling from previous full-order data examples.
- Injecting noise onto previous full-order data examples.

Once collected, we query the policy's expected value for each point in the state space: $\mathbf{u} = \mathbb{E}[\pi_\phi(\mathbf{x})]$ to assemble data $(\mathbf{X}, \mathbf{U})$ and split into the training and validation sets. For each sampling strategy, we use cubic polynomial dictionary and an ensemble of sparse dictionary models using STLRidge using bragging and library bagging. We sweep through the threshold and L^2 penalty hyper-parameters and choose the

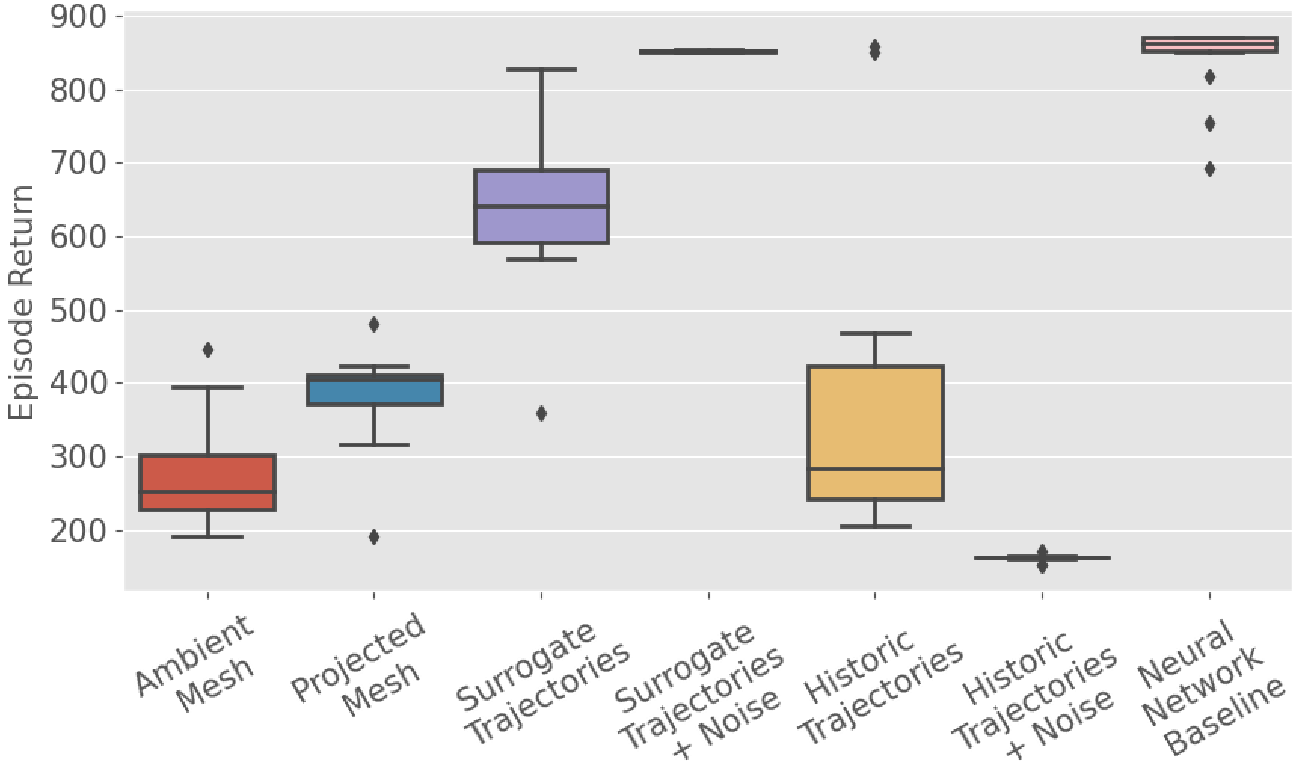

Supplementary Figure 23: Comparison of the six different data collection strategies and the performance of the resulting dictionary model deployed to the full-order environment.

ensemble with minimal validation error. In Figure 9, we show the result of these different sampling strategies by evaluating the learned policy on 15 random initial conditions from the `swing-up` task. By exploiting the surrogate dynamics and learned neural network to produce trajectories, we can greatly increase the desired performance without ever needing to utilize the full-order environment after training. Interestingly, by injecting a small amount of Gaussian noise onto the trajectory before querying the neural network, we do not degrade the performance at all.

7B Swing-up

Figures 24 and 25 demonstrate the effectiveness of the surrogate policy. While the episode returns are larger for the surrogate dictionary, it's important to note that the performance is very comparable; with the median performance only being about 1% different. In Supplementary Figure 26, we highlight a case where we continued to train the `swing-up` policy using Algorithm 1 beyond what was presented in Figures 24 and 25. At some point, the neural network starts to perform worse on the full-order environment, leading to an unstable control policy near the equilibrium. However, when using this neural network to create a surrogate dictionary, the resulting dictionary is a stable, consistent control policy. For both the neural network and the policy, the returns are significantly diminished compared to what's shown in Supplementary Figure 25, and this time the surrogate dictionary's median performance is about 20% better.

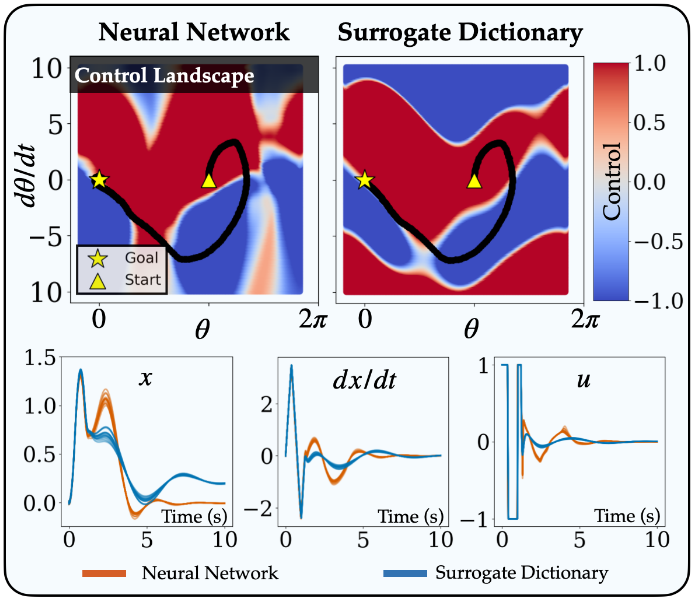

Supplementary Figure 24: Swing-up Policies. Comparison of 15 trajectories from slightly different initial conditions in the full-order environment using the neural network policy (red) and surrogate dictionary approximation (blue). *Top*: The control landscape of the two polices for different $(\theta, d\theta/dt)$ combinations (evaluated at $x = dx/dt = 0$ for ease of visualization) and the corresponding trajectories under the influence of the policies. *Bottom*: The corresponding x, dx/dt, and u for the 15 trajectories.

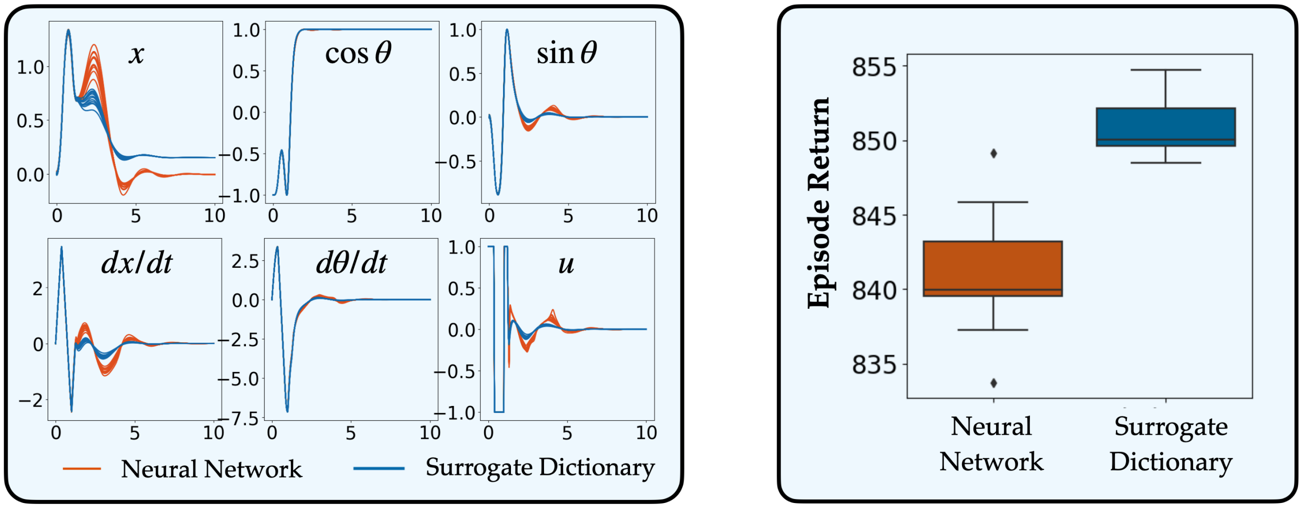

Supplementary Figure 25: Swing-Up Policy: Trajectories. The evaluation of 15 slightly different initial conditions using the surrogate dictionary (blue) and neural network policies discussed in the main text. *Left:* Timeseries of the trajectories and control. *Right:* Distribution of the total cumulative returns from the 15 trajectories.

7C Swimmer-v4

As with the `swing-up` environment, we get a comparable surrogate policy using a cubic dictionary compared to the original neural network, with only a slight difference in median performance. The results can be seen in Supplementary Figure 27.

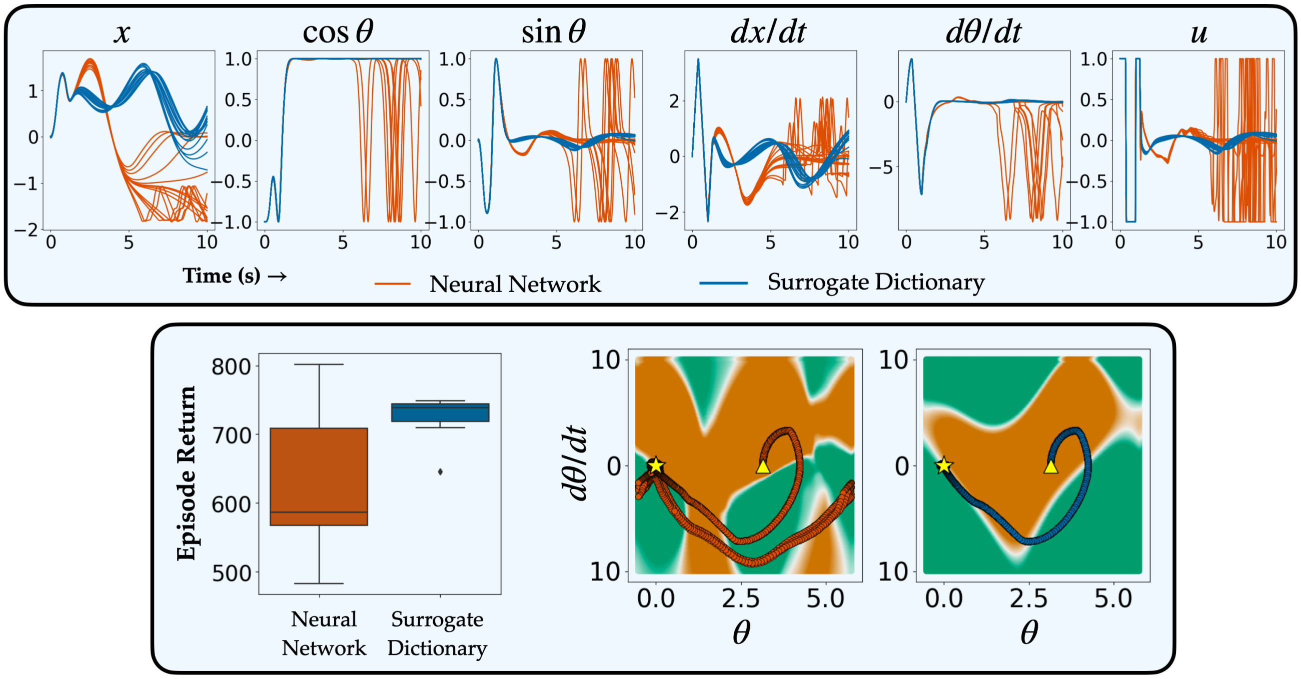

Supplementary Figure 26: Swing-Up Policy: Neural Network Overfitting. The evaluation of 15 slightly different initial conditions using the surrogate dictionary (blue) and neural network policies. *Top:* Timeseries of the trajectories and control. *Bottom Left:* Distribution of the total cumulative returns from the 15 trajectories. *Bottom Right:* The control landscape evaluated on a mesh of $(\theta, \dot{\theta})$ values. (For ease of visualization, $x = \dot{x} = 0$).

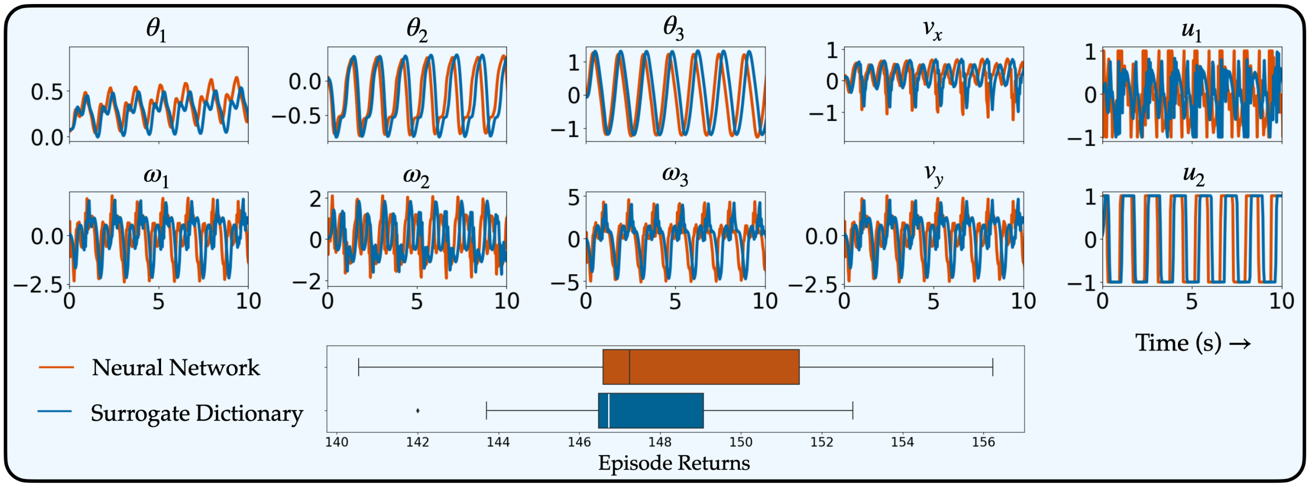

Supplementary Figure 27: Swimmer-v4 Policy Trajectories. Example of one of 15 slightly different initial conditions using the surrogate dictionary (blue) and neural network policies. *Bottom:* Distribution of the total cumulative returns from the 15 trajectories.

7D Cylinder

Unlike the other examples, the neural network Cylinder policy is much more difficult to approximate with a dictionary. As shown in Supplementary Figure 28, when clipping the policies between the maximal control range ($\pm\pi/2$), the neural network policy has essentially learned a "bang-bang" controller. The very thin, nonlinear region of zero-control acts as a discontinuity in the control landscape, which is extremely challenging to approximate with a low-dimensional polynomial. The surrogate dictionary comes close, but has too large of a region where the policy provides small control, leading to the degraded performance.

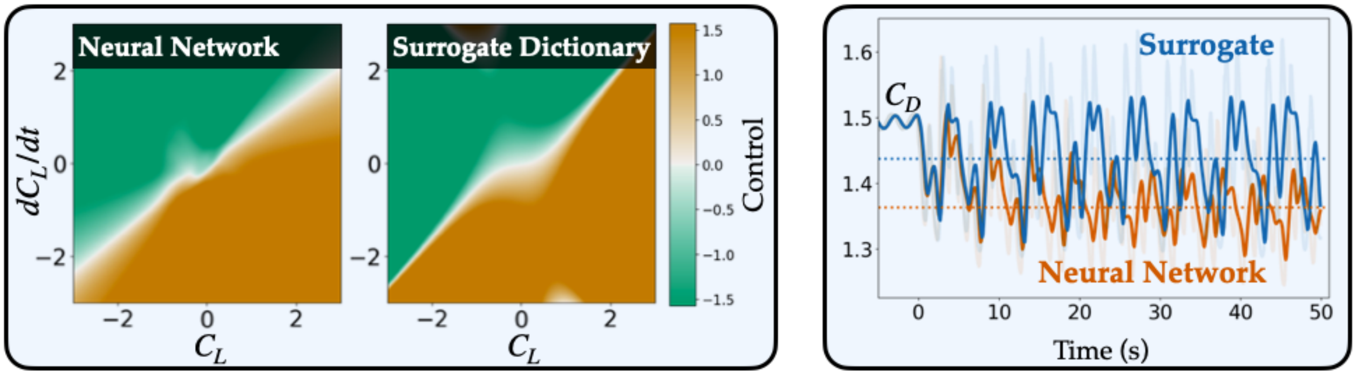

Supplementary Figure 28: Cylinder Policies. *Left*: The control landscapes for the neural network and surrogate policies *Right*: The corresponding full-order C_D for the neural network and surrogate policy evaluation, with control starting at $t = 0$. A 1-second window moving average is plotted in bold. Dotted lines indicate the average C_D over the last 30 seconds.

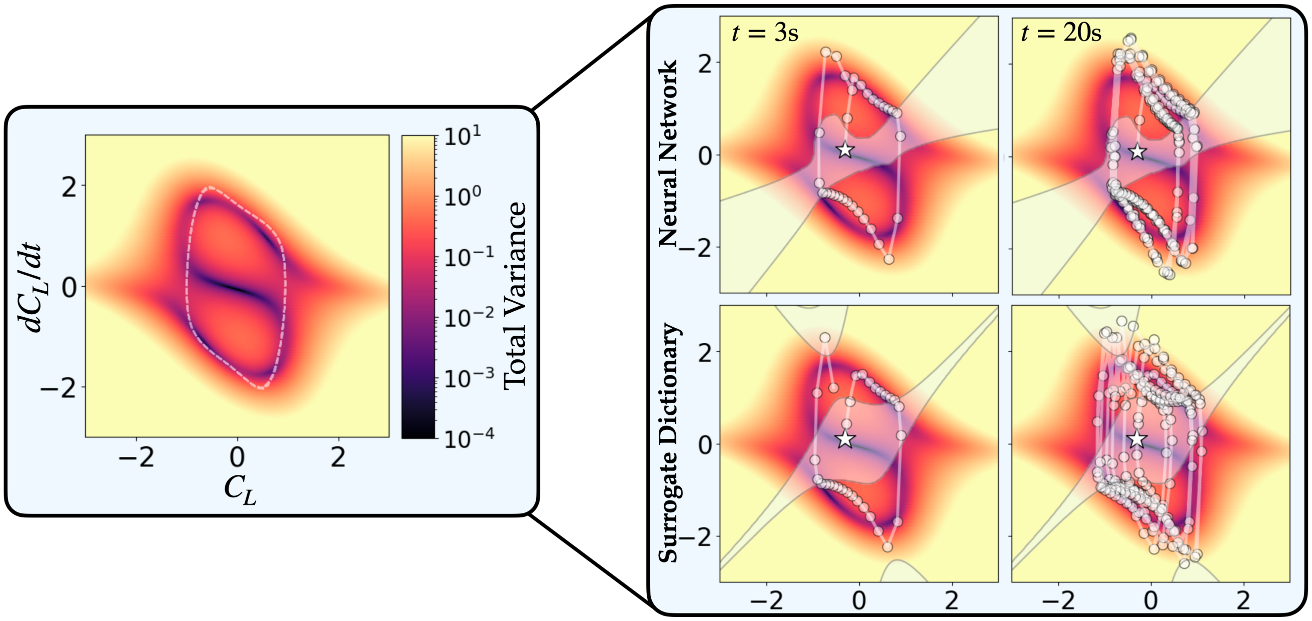

Supplementary Figure 29: Cylinder Policy Variance. *Left:* The variance landscape with the learned limit cycle induced by feedback control in the surrogate environment (dashed white line). *Right:* Snapshots of trajectories from the full order environment produce from the neural network (top) and surrogate policies (bottom) plotted on top of the variance landscape. The star in the center indicate the initial condition, and regions where $|u| < u_{\max} = \pi/2$ (i.e. the light-colored regions from Supplementary Figure 28) are plotted in white for policies.

In Supplementary Figure 29, we inspect the learned policy for the Cylinder agents. There is a low-variance, "s"-shaped basin which is well-aligned with the limit cycle arising from the neural network policy. The data used to fit the surrogate policy was primarily sampled from within the convex hull of the limit cycle; however, when we examine trajectories with feedback control from both policies in the full-order environment, we discover that trajectories leave this limit cycle in both cases. In particular, the dictionary surrogate reaches regions of large variance; at about $t = 3$, it reaches an uncertain region that receives small control input at the top of the basin, bringing it back into a relatively large region of uncertainty on the interior of the limit cycle. The surrogate policy then struggles to reach the limit cycle as it is stuck in large regions of uncertainty. This uncertainty-based analysis hints that additional sampling strategies could reduce the variance of the learned surrogate policy and better imitate the neural network policy.

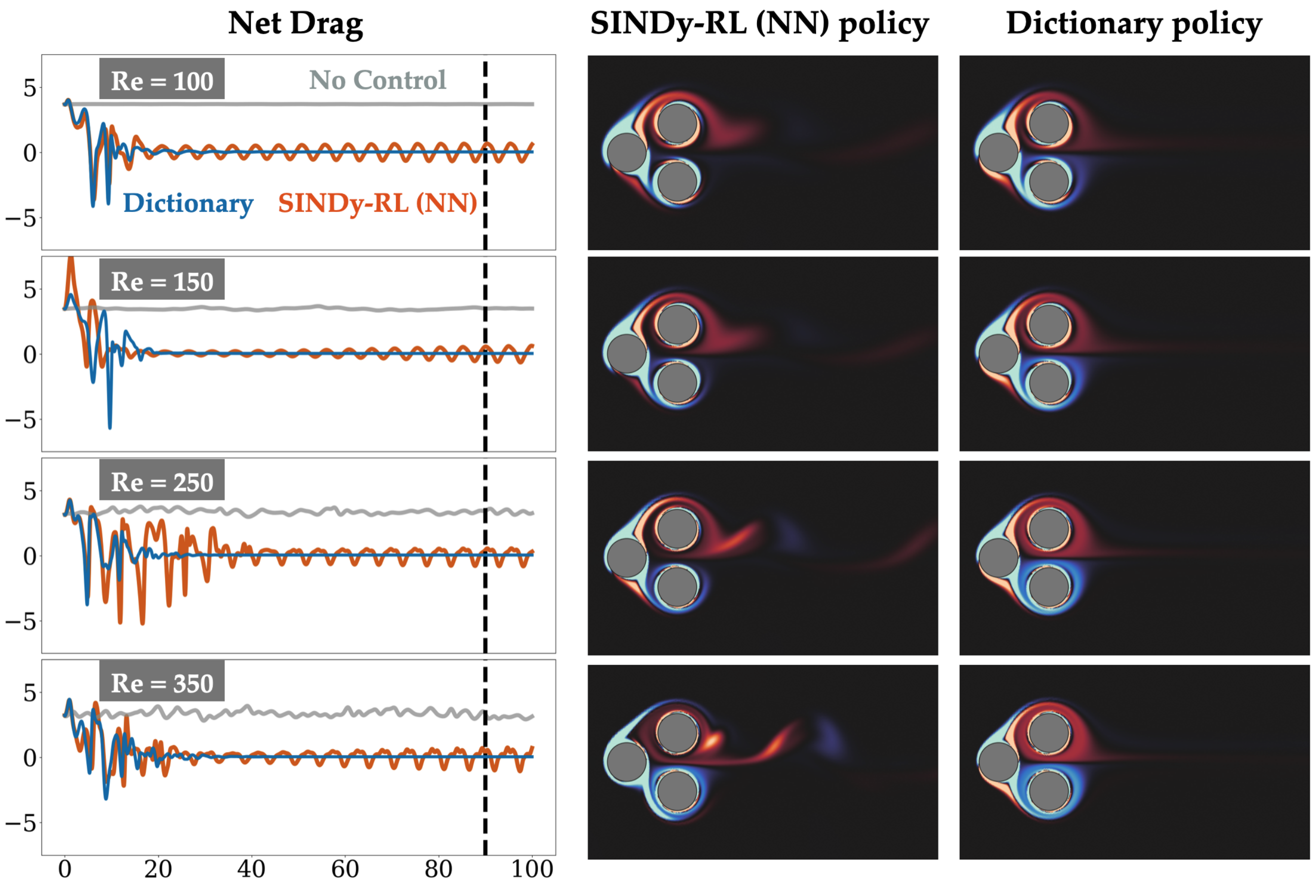

Supplementary Figure 30: Pinball policy Re evaluation. Evaluation of the best performing SINDy-RL NN and corresponding dictionary policy across Re. *Left:* Net drag experienced on the system for the dictionary agents (blue), neural network agents (red), and without control (gray). Negative values indicate thrust generation. Dashed line at $t = 90$ corresponds to the snapshots on the right. *Right:* Vorticity snapshots at $t = 90$.

7E Pinball

In Supplementary Figure 30 we compare the effect of the learned neural network policy obtained from SINDy-RL and the corresponding dictionary policy. Both policies experience an initial transient before settling into stable configuration. Whereas the neural network policy produces small oscillations around zero and small amounts of vortex shedding for large Re, the dictionary policy converges to a fixed drag and stabilizes the wake across all values of Re that we evaluated. In addition to `swing-up` results presented in Supplementary Figure 26, this provides further evidence that dictionary policy might improve the generalization beyond the dynamics it was trained on.

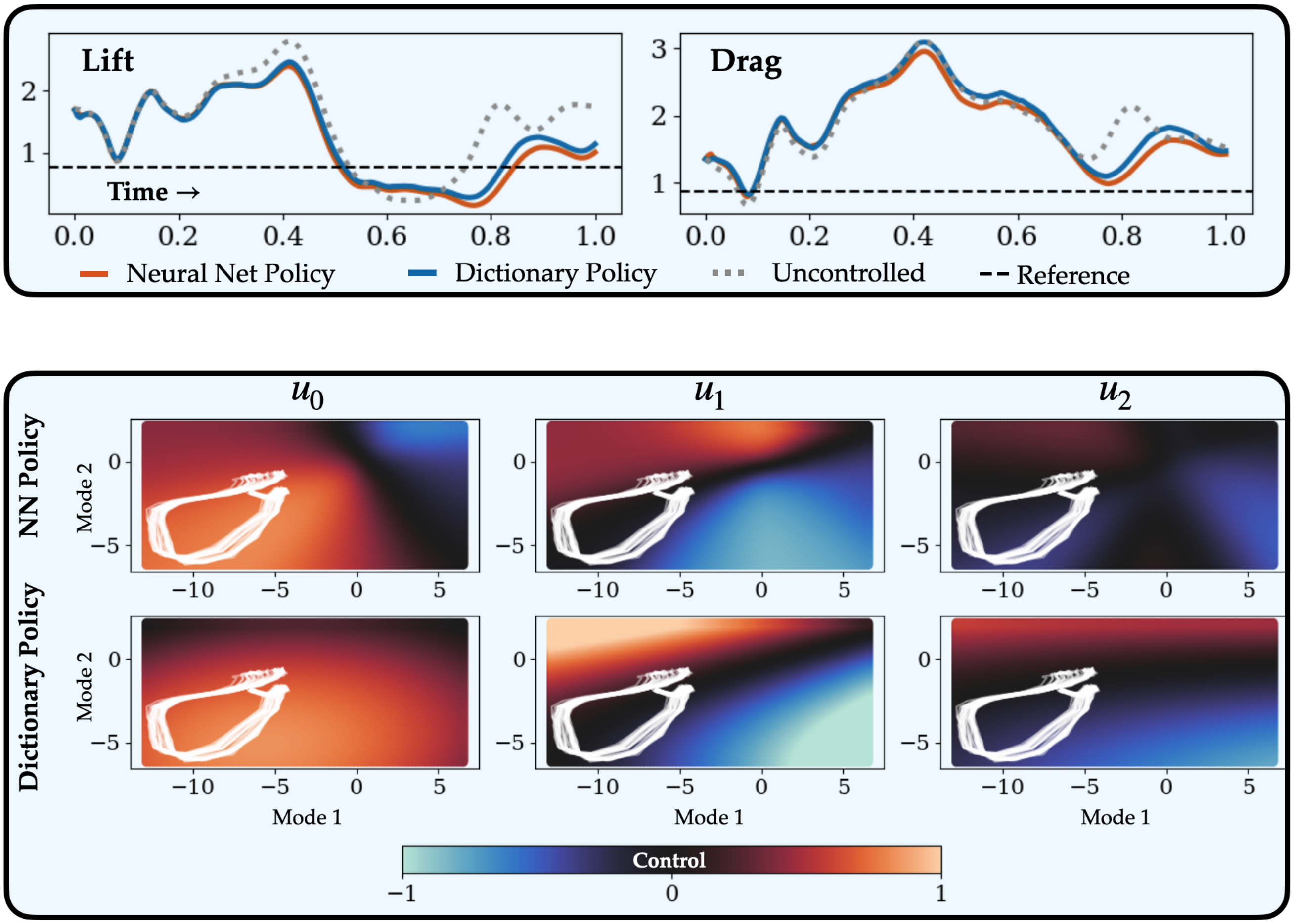

Supplementary Figure 31: 3D Airfoil policy. *Top:* Evaluation of the final SINDy-RL NN and corresponding dictionary policy on the lift and drag forces. The gray dotted line corresponds to the uncontrolled case and the black dashed line corresponds to the desired reference value. *Bottom:* Control landscapes for the neural network and dictionary policies. Trajectories used for training the policy are plotted in white.

7F 3D Airfoil

To approximate the neural network policy obtained by SINDy-RL, a quadratic dictionary was fit:

$$u_0 = 0.501 - 0.022a_1 - 0.108a_2 - 0.003a_1^2 + 0.002a_1a_2 - 0.012a_2^2 \quad (14)$$
$$u_1 = 0.011 - 0.076a_1 + 0.267a_2 - 0.001a_1^2 + 0.001a_1a_2 + 0.021a_2^2 \quad (15)$$
$$u_2 = 0.069 - 0.010a_1 + 0.170a_2 \qquad + 0.003a_1a_2 + 0.009a_2^2 \quad (16)$$
$$(17)$$

Despite its simple form, the mean E-SINDy policy acts as a great approximation of its corresponding neural network policy. The resulting dictionary policy achieved a total return of 24.88, compared to the 25.81 from the neural network. Despite the slightly degraded performance, Supplementary Figure 31 demonstrates the comparable effect of the policy on the lift and drag forces on the airfoil. It can also be qualitatively seen that the dictionary policy finds a slightly smoother control inputs around the on-policy training data.

Supplementary References

[1] Richard Szeliski. *Computer vision: algorithms and applications*. Springer Nature, 2022.

[2] Diksha Khurana, Aditya Koli, Kiran Khatter, and Sukhdev Singh. Natural language processing: State of the art, current trends and challenges. *Multimedia tools and applications*, 82(3):3713–3744, 2023.

[3] Jens Kober and Jan Peters. Reinforcement learning in robotics: A survey. In *Reinforcement Learning*, pages 579–610. Springer, 2012.

[4] Sayon Dutta. *Reinforcement Learning with TensorFlow: A beginner's guide to designing self-learning systems with TensorFlow and OpenAI Gym*. Packt Publishing Ltd, 2018.

[5] Benjamin Recht. A tour of reinforcement learning: The view from continuous control. *Annual Review of Control, Robotics, and Autonomous Systems*, 2:253–279, 2019.

[6] Alekh Agarwal, Nan Jiang, Sham M Kakade, and Wen Sun. Reinforcement learning: Theory and algorithms. *CS Dept., UW Seattle, Seattle, WA, USA, Tech. Rep*, 2019.

[7] S. L. Brunton and J. N. Kutz. *Data-Driven Science and Engineering: Machine Learning, Dynamical Systems, and Control*. Cambridge University Press, 2nd edition, 2022.

[8] Hado Van Hasselt, Arthur Guez, and David Silver. Deep reinforcement learning with double Q-learning. In *Proceedings of the AAAI conference on artificial intelligence*, volume 30, 2016.

[9] Ziyu Wang, Tom Schaul, Matteo Hessel, Hado Hasselt, Marc Lanctot, and Nando Freitas. Dueling network architectures for deep reinforcement learning. In *International conference on machine learning*, pages 1995–2003. PMLR, 2016.

[10] Ahmed H Qureshi, Byron Boots, and Michael C Yip. Adversarial imitation via variational inverse reinforcement learning. *arXiv preprint arXiv:1809.06404*, 2018.

[11] Ching-An Cheng, Xinyan Yan, Nolan Wagener, and Byron Boots. Fast policy learning through imitation and reinforcement. *arXiv preprint arXiv:1805.10413*, 2018.

[12] Volodymyr Mnih, Koray Kavukcuoglu, David Silver, Andrei A Rusu, Joel Veness, Marc G Bellemare, Alex Graves, Martin Riedmiller, Andreas K Fidjeland, Georg Ostrovski, et al. Human-level control through deep reinforcement learning. *Nature*, 518(7540):529, 2015.

[13] David Silver, Julian Schrittwieser, Karen Simonyan, Ioannis Antonoglou, Aja Huang, Arthur Guez, Thomas Hubert, Lucas Baker, Matthew Lai, Adrian Bolton, et al. Mastering the game of Go without human knowledge. *Nature*, 550(7676):354–359, 2017.

[14] David Silver, Thomas Hubert, Julian Schrittwieser, Ioannis Antonoglou, Matthew Lai, Arthur Guez, Marc Lanctot, Laurent Sifre, Dharshan Kumaran, Thore Graepel, et al. A general reinforcement learning algorithm that masters chess, Shogi, and Go through self-play. *Science*, 362(6419):1140–1144, 2018.

[15] Christopher Berner, Greg Brockman, Brooke Chan, Vicki Cheung, Przemysław Debiak, Christy Dennison, David Farhi, Quirin Fischer, Shariq Hashme, Chris Hesse, et al. Dota 2 with large scale deep reinforcement learning. *arXiv preprint arXiv:1912.06680*, 2019.

[16] Oriol Vinyals, Igor Babuschkin, Wojciech M Czarnecki, Michaël Mathieu, Andrew Dudzik, Junyoung Chung, David H Choi, Richard Powell, Timo Ewalds, Petko Georgiev, et al. Grandmaster level in StarCraft ii using multi-agent reinforcement learning. *Nature*, 575(7782):350–354, 2019.

[17] Elia Kaufmann, Leonard Bauersfeld, Antonio Loquercio, Matthias Müller, Vladlen Koltun, and Davide Scaramuzza. Champion-level drone racing using deep reinforcement learning. *Nature*, 620(7976):982–987, 2023.

[18] Jonas Degrave, Federico Felici, Jonas Buchli, Michael Neunert, Brendan Tracey, Francesco Carpanese, Timo Ewalds, Roland Hafner, Abbas Abdolmaleki, Diego de Las Casas, et al. Magnetic control of tokamak plasmas through deep reinforcement learning. *Nature*, 602(7897):414–419, 2022.

[19] Mariya Popova, Olexandr Isayev, and Alexander Tropsha. Deep reinforcement learning for de novo drug design. *Science advances*, 4(7):eaap7885, 2018.

[20] Mattia Gazzola, Babak Hejazialhosseini, and Petros Koumoutsakos. Reinforcement learning and

wavelet adapted vortex methods for simulations of self-propelled swimmers. *SIAM Journal on Scientific Computing*, 36(3):B622–B639, 2014.

[21] Simona Colabrese, Kristian Gustavsson, Antonio Celani, and Luca Biferale. Flow navigation by smart microswimmers via reinforcement learning. *Physical review letters*, 118(15):158004, 2017.

[22] Charles Pivot, Lionel Mathelin, Laurent Cordier, Florimond Guéniat, and Bernd R Noack. A continuous reinforcement learning strategy for closed-loop control in fluid dynamics. In *35th AIAA Applied Aerodynamics Conference*, page 3566, 2017.

[23] Siddhartha Verma, Guido Novati, and Petros Koumoutsakos. Efficient collective swimming by harnessing vortices through deep reinforcement learning. *Proceedings of the National Academy of Sciences*, 115(23):5849–5854, 2018.

[24] Luca Biferale, Fabio Bonaccorso, Michele Buzzicotti, Patricio Clark Di Leoni, and Kristian Gustavsson. Zermelo's problem: Optimal point-to-point navigation in 2d turbulent flows using reinforcement learning. *Chaos: An Interdisciplinary Journal of Nonlinear Science*, 29(10):103138, 2019.

[25] Guido Novati, Lakshminarayanan Mahadevan, and Petros Koumoutsakos. Controlled gliding and perching through deep-reinforcement-learning. *Physical Review Fluids*, 4(9):093902, 2019.

[26] Dixia Fan, Liu Yang, Zhicheng Wang, Michael S Triantafyllou, and George Em Karniadakis. Reinforcement learning for bluff body active flow control in experiments and simulations. *Proceedings of the National Academy of Sciences*, 117(42):26091–26098, 2020.

[27] J. Rabault and A. Kuhnle. *Deep Reinforcement Learning Applied to Active Flow Control*, page 368–390. Cambridge University Press, 2023.

[28] Gerben Beintema, Alessandro Corbetta, Luca Biferale, and Federico Toschi. Controlling rayleigh-bénard convection via reinforcement learning. *arXiv preprint arXiv:2003.14358*, 2020.

[29] Guido Novati, Hugues Lascombes de Laroussilhe, and Petros Koumoutsakos. Automating turbulence modelling by multi-agent reinforcement learning. *Nature Machine Intelligence*, 3(1):87–96, 2021.

[30] H Jane Bae and Petros Koumoutsakos. Scientific multi-agent reinforcement learning for wall-models of turbulent flows. *Nature Communications*, 13(1):1443, 2022.

[31] Sergey Levine, Aviral Kumar, George Tucker, and Justin Fu. Offline reinforcement learning: Tutorial, review, and perspectives on open problems. *arXiv preprint arXiv:2005.01643*, 2020.

[32] Ahmed Hussein, Mohamed Medhat Gaber, Eyad Elyan, and Chrisina Jayne. Imitation learning: A survey of learning methods. *ACM Computing Surveys (CSUR)*, 50(2):1–35, 2017.

[33] Bin Fang, Shidong Jia, Di Guo, Muhua Xu, Shuhuan Wen, and Fuchun Sun. Survey of imitation learning for robotic manipulation. *International Journal of Intelligent Robotics and Applications*, 3:362–369, 2019.

[34] Luc Le Mero, Dewei Yi, Mehrdad Dianati, and Alexandros Mouzakitis. A survey on imitation learning techniques for end-to-end autonomous vehicles. *IEEE Transactions on Intelligent Transportation Systems*, 23(9):14128–14147, 2022.

[35] Tom Schaul, John Quan, Ioannis Antonoglou, and David Silver. Prioritized experience replay. *Proceedings of the International Conference on Learning Representations*, 2016.

[36] David Rolnick, Arun Ahuja, Jonathan Schwarz, Timothy Lillicrap, and Gregory Wayne. Experience replay for continual learning. *Advances in Neural Information Processing Systems*, 32, 2019.

[37] Marcin Andrychowicz, Filip Wolski, Alex Ray, Jonas Schneider, Rachel Fong, Peter Welinder, Bob McGrew, Josh Tobin, OpenAI Pieter Abbeel, and Wojciech Zaremba. Hindsight experience replay. *Advances in neural information processing systems*, 30, 2017.

[38] Zhuangdi Zhu, Kaixiang Lin, Anil K Jain, and Jiayu Zhou. Transfer learning in deep reinforcement learning: A survey. *IEEE Transactions on Pattern Analysis and Machine Intelligence*, 2023.

[39] Jane X Wang, Zeb Kurth-Nelson, Dhruva Tirumala, Hubert Soyer, Joel Z Leibo, Remi Munos, Charles Blundell, Dharshan Kumaran, and Matt Botvinick. Learning to reinforcement learn. *arXiv preprint arXiv:1611.05763*, 2016.

[40] Chelsea Finn, Pieter Abbeel, and Sergey Levine. Model-agnostic meta-learning for fast adaptation of

deep networks. In *International conference on machine learning*, pages 1126–1135. PMLR, 2017.

[41] Sebastian Höfer, Kostas Bekris, Ankur Handa, Juan Camilo Gamboa, Melissa Mozifian, Florian Golemo, Chris Atkeson, Dieter Fox, Ken Goldberg, John Leonard, et al. Sim2real in robotics and automation: Applications and challenges. *IEEE transactions on automation science and engineering*, 18(2):398–400, 2021.

[42] Frank L Lewis, Draguna Vrabie, and Vassilis L Syrmos. *Optimal control*. John Wiley & Sons, 2012.

[43] Lars Grüne, Jürgen Pannek, Lars Grüne, and Jürgen Pannek. *Nonlinear model predictive control*. Springer, 2017.

[44] Richard S Sutton. Integrated architectures for learning, planning, and reacting based on approximating dynamic programming. In *Machine learning proceedings 1990*, pages 216–224. Elsevier, 1990.

[45] Tingwu Wang, Xuchan Bao, Ignasi Clavera, Jerrick Hoang, Yeming Wen, Eric Langlois, Shunshi Zhang, Guodong Zhang, Pieter Abbeel, and Jimmy Ba. Benchmarking model-based reinforcement learning. *arXiv preprint arXiv:1907.02057*, 2019.

[46] Ignasi Clavera, Jonas Rothfuss, John Schulman, Yasuhiro Fujita, Tamim Asfour, and Pieter Abbeel. Model-based reinforcement learning via meta-policy optimization. In *Conference on Robot Learning*, pages 617–629. PMLR, 2018.

[47] Steven L Brunton, Joshua L Proctor, and J Nathan Kutz. Discovering governing equations from data by sparse identification of nonlinear dynamical systems. *Proceedings of the National Academy of Sciences*, 113(15):3932–3937, 2016.

[48] Mariia Sorokina, Stylianos Sygletos, and Sergei Turitsyn. Sparse identification for nonlinear optical communication systems: SINO method. *Optics express*, 24(26):30433–30443, 2016.

[49] Letetia Mary Addison. Dynamics of a prey-predator model using sparse identification: A data driven approach. *Journal of Coupled Systems and Multiscale Dynamics*, 5(2):143–150, 2017.

[50] Magnus Dam, Morten Brøns, Jens Juul Rasmussen, Volker Naulin, and Jan S Hesthaven. Sparse identification of a predator-prey system from simulation data of a convection model. *Physics of Plasmas*, 24(2):022310, 2017.

[51] Linan Zhang and Hayden Schaeffer. On the convergence of the sindy algorithm. *Multiscale Modeling & Simulation*, 17(3):948–972, 2019.

[52] Lorenzo Boninsegna, Feliks Nüske, and Cecilia Clementi. Sparse learning of stochastic dynamical equations. *The Journal of Chemical Physics*, 148(24):241723, 2018.

[53] Stephan Thaler, Ludger Paehler, and Nikolaus A Adams. Sparse identification of truncation errors. *Journal of Computational Physics*, 397:108851, 2019.

[54] Wei Zhang, Stefan Klus, Tim Conrad, and Christof Schütte. Learning chemical reaction networks from trajectory data. *SIAM Journal on Applied Dynamical Systems*, 18(4):2000–2046, 2019.

[55] J.-C. Loiseau and S. L. Brunton. Constrained sparse Galerkin regression. *Journal of Fluid Mechanics*, 838:42–67, 2018.

[56] J.-C. Loiseau, B. R. Noack, and S. L. Brunton. Sparse reduced-order modeling: sensor-based dynamics to full-state estimation. *Journal of Fluid Mechanics*, 844:459–490, 2018.

[57] Jared L Callaham, J-C Loiseau, Georgios Rigas, and Steven L Brunton. Nonlinear stochastic modelling with Langevin regression. *Proceedings of the Royal Society A*, 477(2250):20210092, 2021.

[58] Yifei Guan, Steven L Brunton, and Igor Novosselov. Sparse nonlinear models of chaotic electroconvection. *Royal Society Open Science*, 8(8):202367, 2021.

[59] Jean-Christophe Loiseau. Data-driven modeling of the chaotic thermal convection in an annular thermosyphon. *Theoretical and Computational Fluid Dynamics*, 34, 2020.

[60] N. Deng, B. R. Noack, M. Morzynski, and L. R. Pastur. Low-order model for successive bifurcations of the fluidic pinball. *Journal of Fluid Mechanics*, 884(A37), 2020.

[61] Nan Deng, Bernd R Noack, Marek Morzyński, and Luc R Pastur. Galerkin force model for transient and post-transient dynamics of the fluidic pinball. *Journal of Fluid Mechanics*, 918, 2021.

[62] Jared L Callaham, Steven L Brunton, and Jean-Christophe Loiseau. On the role of nonlinear correlations in reduced-order modeling. *Journal of Fluid Mechanics*, 938(A1), 2022.

[63] Jared L Callaham, Georgios Rigas, Jean-Christophe Loiseau, and Steven L Brunton. An empirical mean-field model of symmetry-breaking in a turbulent wake. *Science Advances*, 8(eabm4786), 2022.

[64] Laure Zanna and Thomas Bolton. Data-driven equation discovery of ocean mesoscale closures. *Geophysical Research Letters*, 47(17):e2020GL088376, 2020.

[65] Martin Schmelzer, Richard P Dwight, and Paola Cinnella. Discovery of algebraic Reynolds-stress models using sparse symbolic regression. *Flow, Turbulence and Combustion*, 104(2):579–603, 2020.

[66] S Beetham and J Capecelatro. Formulating turbulence closures using sparse regression with embedded form invariance. *Physical Review Fluids*, 5(8):084611, 2020.

[67] Sarah Beetham, Rodney O Fox, and Jesse Capecelatro. Sparse identification of multiphase turbulence closures for coupled fluid–particle flows. *Journal of Fluid Mechanics*, 914, 2021.

[68] S. H. Rudy, S. L. Brunton, J. L. Proctor, and J. N. Kutz. Data-driven discovery of partial differential equations. *Science Advances*, 3(e1602614), 2017.

[69] K. Champion, B. Lusch, J. Nathan Kutz, and Steven L. Brunton. Data-driven discovery of coordinates and governing equations. *Proceedings of the National Academy of Sciences*, 116(45):22445–22451, 2019.

[70] Alan A Kaptanoglu, Jared L Callaham, Christopher J Hansen, Aleksandr Aravkin, and Steven L Brunton. Promoting global stability in data-driven models of quadratic nonlinear dynamics. *Physical Review Fluids*, 6(094401), 2021.

[71] Hayden Schaeffer and Scott G McCalla. Sparse model selection via integral terms. *Physical Review E*, 96(2):023302, 2017.

[72] Patrick AK Reinbold, Daniel R Gurevich, and Roman O Grigoriev. Using noisy or incomplete data to discover models of spatiotemporal dynamics. *Physical Review E*, 101(1):010203, 2020.

[73] Daniel A Messenger and David M Bortz. Weak SINDy for partial differential equations. *Journal of Computational Physics*, 443:110525, 2021.

[74] Daniel A Messenger and David M Bortz. Weak SINDy: Galerkin-based data-driven model selection. *Multiscale Modeling & Simulation*, 19(3):1474–1497, 2021.

[75] Gert-Jan Both, Subham Choudhury, Pierre Sens, and Remy Kusters. Deepmod: Deep learning for model discovery in noisy data. *Journal of Computational Physics*, 428:109985, 2021.

[76] Ali Forootani, Pawan Goyal, and Peter Benner. A robust sindy approach by combining neural networks and an integral form. *arXiv preprint arXiv:2309.07193*, 2023.

[77] Urban Fasel, J Nathan Kutz, Bingni W Brunton, and Steven L Brunton. Ensemble-SINDy: Robust sparse model discovery in the low-data, high-noise limit, with active learning and control. *Proceedings of the Royal Society A*, 478(2260):20210904, 2022.

[78] Robert K Niven, Ali Mohammad-Djafari, Laurent Cordier, Markus Abel, and Markus Quade. Bayesian identification of dynamical systems. *Multidisciplinary Digital Publishing Institute Proceedings*, 33(1):33, 2020.

[79] L Gao, Urban Fasel, Steven L Brunton, and J Nathan Kutz. Convergence of uncertainty estimates in ensemble and Bayesian sparse model discovery. *arXiv preprint arXiv:2301.12649*, 2023.

[80] Steven L Brunton, Joshua L Proctor, and J Nathan Kutz. Sparse identification of nonlinear dynamics with control (SINDYc). *IFAC-PapersOnLine*, 49(18):710–715, 2016.

[81] Eurika Kaiser, J Nathan Kutz, and Steven L Brunton. Sparse identification of nonlinear dynamics for model predictive control in the low-data limit. *Proceedings of the Royal Society A*, 474(2219):20180335, 2018.

[82] Urban Fasel, Eurika Kaiser, J Nathan Kutz, Bingni W Brunton, and Steven L Brunton. SINDy with control: A tutorial. In *2021 60th IEEE Conference on Decision and Control (CDC)*, pages 16–21. IEEE, 2021.

[83] JD Lore, S De Pascuale, P Laiu, B Russo, J-S Park, JM Park, SL Brunton, JN Kutz, and AA Kaptanoglu. Time-dependent SOLPS-ITER simulations of the tokamak plasma boundary for model predictive control

using SINDy. *Nuclear Fusion*, 63(4):046015, 2023.

[84] Milad Farsi and Jun Liu. Structured online learning-based control of continuous-time nonlinear systems. *IFAC-PapersOnLine*, 53(2):8142–8149, 2020.

[85] Milad Farsi and Jun Liu. A structured online learning approach to nonlinear tracking with unknown dynamics. In *2021 American Control Conference (ACC)*, pages 2205–2211. IEEE, 2021.

[86] Rushiv Arora, Bruno Castro da Silva, and Eliot Moss. Model-based reinforcement learning with SINDy. *arXiv preprint arXiv:2208.14501*, 2022.

[87] Richard S Sutton and Andrew G Barto. *Reinforcement learning: An introduction*, volume 1. MIT press Cambridge, 1998.

[88] Anil V Rao. A survey of numerical methods for optimal control. *Advances in the astronautical Sciences*, 135(1):497–528, 2009.

[89] Anusha Nagabandi, Gregory Kahn, Ronald S Fearing, and Sergey Levine. Neural network dynamics for model-based deep reinforcement learning with model-free fine-tuning. In *2018 IEEE international conference on robotics and automation (ICRA)*, pages 7559–7566. IEEE, 2018.

[90] Kurtland Chua, Roberto Calandra, Rowan McAllister, and Sergey Levine. Deep reinforcement learning in a handful of trials using probabilistic dynamics models. *Advances in neural information processing systems*, 31, 2018.

[91] Marc Deisenroth and Carl E Rasmussen. Pilco: A model-based and data-efficient approach to policy search. In *Proceedings of the 28th International Conference on machine learning (ICML-11)*, pages 465–472, 2011.

[92] Yuval Tassa, Tom Erez, and Emanuel Todorov. Synthesis and stabilization of complex behaviors through online trajectory optimization. In *2012 IEEE/RSJ International Conference on Intelligent Robots and Systems*, pages 4906–4913. IEEE, 2012.

[93] Nicolas Heess, Gregory Wayne, David Silver, Timothy Lillicrap, Tom Erez, and Yuval Tassa. Learning continuous control policies by stochastic value gradients. *Advances in neural information processing systems*, 28, 2015.

[94] Sergey Levine, Chelsea Finn, Trevor Darrell, and Pieter Abbeel. End-to-end training of deep visuomotor policies. *The Journal of Machine Learning Research*, 17(1):1334–1373, 2016.

[95] Richard S Sutton, Csaba Szepesvári, Alborz Geramifard, and Michael P Bowling. Dyna-style planning with linear function approximation and prioritized sweeping. *Proceedings of the Twenty-Fourth Conference on Uncertainty in Artificial Intelligence*, 2008.

[96] Matthew Retchin, Brandon Amos, Steven L. Brunton, and Shuran Song. Koopman constrained policy optimization: A Koopman operator theoretic method for differentiable optimal control in robotics. In *ICML 2023 Workshop on Differentiable Almost Everything: Differentiable Relaxations, Algorithms, Operators, and Simulators*, 2023.

[97] P. Rozwood, E. Mehrez, L. Paehler, and S. L. Brunton. Koopman assisted reinforcement learning. In *NeurIPS Workshop on AI4Science*, 2023.

[98] Josh Bongard and Hod Lipson. Automated reverse engineering of nonlinear dynamical systems. *Proceedings of the National Academy of Sciences*, 104(24):9943–9948, 2007.

[99] Michael Schmidt and Hod Lipson. Distilling free-form natural laws from experimental data. *Science*, 324(5923):81–85, 2009.

[100] Miles Cranmer. Interpretable machine learning for science with pysr and symbolicregression. jl. *arXiv preprint arXiv:2305.01582*, 2023.

[101] Robert Tibshirani. Regression shrinkage and selection via the lasso. *Journal of the Royal Statistical Society. Series B (Methodological)*, pages 267–288, 1996.

[102] Kadierdan Kaheman, J Nathan Kutz, and Steven L Brunton. SINDy-PI: a robust algorithm for parallel implicit sparse identification of nonlinear dynamics. *Proceedings of the Royal Society A*, 476(2242):20200279, 2020.

[103] Saran Tunyasuvunakool, Alistair Muldal, Yotam Doron, Siqi Liu, Steven Bohez, Josh Merel, Tom Erez, Timothy Lillicrap, Nicolas Heess, and Yuval Tassa. dm_control: Software and tasks for continuous control. *Software Impacts*, 6:100022, 2020.

[104] Jiayi Weng, Huayu Chen, Dong Yan, Kaichao You, Alexis Duburcq, Minghao Zhang, Yi Su, Hang Su, and Jun Zhu. Tianshou: A highly modularized deep reinforcement learning library. *The Journal of Machine Learning Research*, 23(1):12275–12280, 2022.

[105] Shengyi Huang, Rousslan Fernand Julien Dossa, Chang Ye, Jeff Braga, Dipam Chakraborty, Kinal Mehta, and João G.M. Araújo. Cleanrl: High-quality single-file implementations of deep reinforcement learning algorithms. *Journal of Machine Learning Research*, 23(274):1–18, 2022.

[106] Maël Franceschetti, Coline Lacoux, Ryan Ohouens, Antonin Raffin, and Olivier Sigaud. Making reinforcement learning work on swimmer. *arXiv preprint arXiv:2208.07587*, 2022.

[107] Pau Varela, Pol Suárez, Francisco Alcántara-Ávila, Arnau Miró, Jean Rabault, Bernat Font, Luis Miguel García-Cuevas, Oriol Lehmkuhl, and Ricardo Vinuesa. Deep reinforcement learning for flow control exploits different physics for increasing reynolds number regimes. In *Actuators*, volume 11, page 359. MDPI, 2022.

[108] Qiulei Wang, Lei Yan, Gang Hu, Wenli Chen, Jean Rabault, and Bernd R Noack. Dynamic feature-based deep reinforcement learning for flow control of circular cylinder with sparse surface pressure sensing. *arXiv preprint arXiv:2307.01995*, 2023.

[109] BR Noack and M Morzynski. The fluidic pinball: A toolkit for multiple-input multiple-output flow control. Technical report, Tech. Rep. 02, 2017.

[110] Nan Deng, Bernd R Noack, Marek Morzyński, and Luc R Pastur. Low-order model for successive bifurcations of the fluidic pinball. *Journal of fluid mechanics*, 884:A37, 2020.

[111] Andreas Lintermann, Matthias Meinke, and Wolfgang Schröder. Zonal flow solver (zfs): a highly efficient multi-physics simulation framework. *International Journal of Computational Fluid Dynamics*, 34(7-8):458–485, 2020.

[112] Christian Lagemann, Mario Rüttgers, Miro Gondrum, Matthias Meinke, Wolfgang Schröder, Andreas Lintermann, and Steven L. Brunton. Hydrogym-gpu: From 2d to 3d benchmark environments for reinforcement learning in fluid flows. In *Proceedings of the 35th International Conference on Parallel Computational Fluid Dynamics (ParCFD2024)*, Bonn, Germany, September 2024.

[113] Andreas Lintermann, Stefan Schlimpert, Johannes Grimmen, Christoph Günther, Matthias Meinke, and Wolfgang Schröder. Massively parallel grid generation on hpc systems. *Computer Methods in Applied Mechanics and Engineering*, 277:131–153, 2014.

[114] Xiaoyi He and Li-Shi Luo. Theory of the lattice boltzmann method: From the boltzmann equation to the lattice boltzmann equation. *Physical Review E*, 56(6):6811–6817, 1997.

[115] Martin Geier, Martin Schönherr, Andrea Pasquali, and Manfred Krafczyk. The cumulant lattice boltzmann equation in three dimensions: Theory and validation. *Computers & Mathematics with Applications*, 70(4):507–547, 2015.

[116] Salvador Izquierdo and Norberto Fueyo. Characteristic nonreflecting boundary conditions for open boundaries in lattice boltzmann methods. *Physical Review E*, 78(4):046707, 2008.

[117] Taiba Kouser, Yongliang Xiong, Dan Yang, and Sai Peng. Direct numerical simulations on the three-dimensional wake transition of flows over naca0012 airfoil at re= 1000. *International Journal of Micro Air Vehicles*, 13:17568293211055656, 2021.

[118] Yannick Hoarau, Marianna Braza, Y Ventikos, and D Faghani. First stages of the transition to turbulence and control in the incompressible detached flow around a naca0012 wing. *International journal of heat and fluid flow*, 27(5):878–886, 2006.

[119] Eric Liang, Richard Liaw, Robert Nishihara, Philipp Moritz, Roy Fox, Ken Goldberg, Joseph Gonzalez, Michael Jordan, and Ion Stoica. RLlib: Abstractions for distributed reinforcement learning. In Jennifer Dy and Andreas Krause, editors, *Proceedings of the 35th International Conference on Machine Learning*, volume 80 of *Proceedings of Machine Learning Research*, pages 3053–3062. PMLR, 10–15 Jul 2018.

[120] John Schulman, Filip Wolski, Prafulla Dhariwal, Alec Radford, and Oleg Klimov. Proximal policy optimization algorithms. *arXiv preprint arXiv:1707.06347*, 2017.

[121] Richard Liaw, Eric Liang, Robert Nishihara, Philipp Moritz, Joseph E Gonzalez, and Ion Stoica. Tune: A research platform for distributed model selection and training. *arXiv preprint arXiv:1807.05118*, 2018.

[122] Brian de Silva, Kathleen Champion, Markus Quade, Jean-Christophe Loiseau, J. Kutz, and Steven Brunton. PySINDy: A python package for the sparse identification of nonlinear dynamical systems from data. *Journal of Open Source Software*, 5(49):2104, 2020.

[123] Alan A. Kaptanoglu, Brian M. de Silva, Urban Fasel, Kadierdan Kaheman, Andy J. Goldschmidt, Jared Callaham, Charles B. Delahunt, Zachary G. Nicolaou, Kathleen Champion, Jean-Christophe Loiseau, J. Nathan Kutz, and Steven L. Brunton. PySINDy: A comprehensive python package for robust sparse system identification. *Journal of Open Source Software*, 7(69):3994, 2022.
